# Enhanced Aquila optimizer based on tent chaotic mapping and new rules

**DOI:** 10.1038/s41598-024-53064-6

**Published:** 2024-02-06

**Authors:** Youfa Fu, Dan Liu, Shengwei Fu, Jiadui Chen, Ling He

**Affiliations:** Key Laboratory of Advanced Manufacturing Technology, Ministry of Education, Guizhou University, Guiyang, 550025 Guizhou China

**Keywords:** Computational science, Computer science

## Abstract

Metaheuristic algorithms, widely applied across various domains due to their simplicity and strong optimization capabilities, play a crucial role in problem-solving. While the Aquila Optimizer is recognized for its effectiveness, it often exhibits slow convergence rates and susceptibility to local optima in certain scenarios. To address these concerns, this paper introduces an enhanced version, termed Tent-enhanced Aquila Optimizer (TEAO). TEAO incorporates the Tent chaotic map to initialize the Aquila population, promoting a more uniform distribution within the solution space. To balance exploration and exploitation, novel formulas are proposed, accelerating convergence while ensuring precision. The effectiveness of the TEAO algorithm is validated through a comprehensive comparison with 14 state-of-the-art algorithms using 23 classical benchmark test functions. Additionally, to assess the practical feasibility of the approach, TEAO is applied to six constrained engineering problems and benchmarked against the performance of the same 14 algorithms. All experimental results consistently demonstrate that TEAO outperforms other advanced algorithms in terms of solution quality and stability, establishing it as a more competitive choice for optimization tasks.

## Introduction

The optimization process aims to identify points that satisfy all the constraints and bring the objective function as close as possible to the minimum (or maximum) among a large number of points. The goal is to discover the optimal decision variables for the system by minimizing the cost function among all possible values^1^. In various fields, including image processing^2^, engineering problems^3^, wireless sensor networks^4^, scheduling problems^5,6^, path planning^7^, feature selection^8^, data clustering^9^, optimization problems are crucial. Practical engineering problems exhibit characteristics such as nonlinearity, extensive calculations, and a broad solution space. Traditional optimization methods, due to the gradient mechanism, suffer from drawbacks such as poor flexibility, high computational complexity, and a tendency to fall into local optimal solutions. To address these shortcomings, numerous meta-heuristic (MH) algorithms have been proposed. Meta-heuristic (MH) algorithms excel in optimization problems, offering high flexibility, no gradient mechanism, and a strong ability to escape local optimal trap^10^. Meta-heuristic algorithms (MH) have been widely promoted.

The study of metaheuristic algorithms for solving optimization problems has reached a critical stage. These algorithms often leverage biological or physical phenomena to discover solutions for real-world optimization problems. Metaheuristic algorithms use random processes to generate feasible solution spaces, perform solution searches during each iteration, assess individual fitness through a fitness function, and update to create the best possible solution^11^. Based on their sources of inspiration, metaheuristic algorithms can be classified into four categories^12^: evolutionary algorithms (EA), physical algorithms (PhA), swarm intelligence (SI) algorithms, and human-based algorithms. Despite their distinctive sources of inspiration, all these algorithms go through two important stages in their search process: exploration and exploitation^13,14^. The exploration stage involves a comprehensive and efficient search of the solution space, enabling the algorithm to avoid local optima, while the exploitation stage aims to enhance local quality and find optimal solutions within the obtained solution space. Therefore, a well-designed metaheuristic (MH) algorithm should strike a balance between these two stages, ensuring optimal performance.

Swarm Intelligence (SI) is a category of metaheuristic algorithms that has rapidly developed in recent decades. Based on social behavior observed in various biological groups, such as ants, bats, whales, birds, and wolves, SI is employed to optimize solutions. One example of an SI algorithm is particle swarm optimization (PSO), proposed by Eberhart et al.^15^, where each particle represents a candidate solution and is updated based on both the global best position and its local position. Another example is the artificial bee colony algorithm (ABC) proposed by Karaboga et al.^16^, which is based on the division of labor and cooperative behavior observed in bee colonies. Evolutionary algorithms (EA)^17^ are probabilistic optimization algorithms inspired by natural evolution rules, such as the genetic algorithm (GA) proposed by Holland^18^, grounded in Darwin's theory of evolution. Population optimization algorithms inspired by physical phenomena and laws, such as the gravitational search algorithm (GSA) by Rashedi et al.^19^, are referred to as Physical-based algorithms (PhA). Human-based algorithms are inspired by human behavior, such as the social evolution and learning optimization algorithm (SELOA) proposed by Kumar et al.^20^, grounded in social learning behavior. The Learner Performance-Based Behavior Algorithm^21^ proposed by Rahman is inspired by the process of admitting high school graduates from various academic disciplines into university programs and the changes learners undergo in their study behaviors to enhance academic performance. This article mainly introduces the swarm intelligence algorithm and its applications.

The particle swarm optimization (PSO) algorithm, initially introduced by Eberhart et al.^15^, has found widespread applications in solving various problems, including global optimization, document classification, image segmentation, feature selection^22,23^, data clustering, scheduling problems^24^, and other industrial and engineering challenges^25^. Similarly, the ant colony optimization algorithm (ACO), proposed by Dorigo et al.^26^, draws inspiration from the social behavior of ant colony foraging. Ants use pheromones to mark favorable paths for their colony members. The algorithm based on this behavior has garnered extensive research attention and has been applied to various optimization problems, including feature selection, path planning, data mining, classification, and time series prediction^27,28^. The Firefly Algorithm (FA), proposed by Yang et al.^29^, was utilized by Shafiei et al.^30^ to model the membership function of the Adaptive Neuro-Fuzzy Inference System (ANFIS) and the discharge coefficient of labyrinth weir. In 2014, Mirjalili et al.^31^ proposed the Grey Wolf Optimization Algorithm (GWO), and in 2013, Gandomi et al.^32^ introduced the Cuckoo Search Algorithm (CS). Furthermore, in 2016, Mirjalili et al.^33^ proposed the Whale Optimization Algorithm (WOA). Additionally, the Salp Group Algorithm (SSA), inspired by the group behavior of salps during foraging and parading in the ocean, was proposed by Mirjalili et al.^34^ in 2017. The Remora optimization algorithm (ROA), proposed by Jia et al.^35^ in 2020, is inspired by the foraging and diffusion behavior of slime mold. The Horse Swarm Optimization Algorithm (HOA) proposed by MiarNaimi et al.^36^ in 2021. Inspired by the search for the best food area or prey in the collective action of animal groups, Yapici et al.^37^ proposed the Pathfinder Algorithm (PFA). The main inspiration for MPA^38^ comes from the foraging behaviour of marine predators. The chameleon swarm algorithm (CSA), proposed by Braik^39^, is inspired by the dynamic behavior of chameleons when searching for food near trees, deserts, and swamps. The main inspiration for the DA algorithm^40^ comes from the static and dynamic gregarious behavior of dragonflies in nature, The Artificial Gorilla Force Optimization (GTO)^41^ is inspired by the social behavior of gorilla groups. The squirrel search algorithm (SSA) is a new natural heuristic algorithm proposed by Jain et al.^42^ based on the dynamic foraging and gliding behavior of squirrels. The coyote optimization algorithm (COA) proposed by Pierezan and Coelho^43^, is a new meta-heuristic algorithm for solving global optimization problems, inspired by the canine class. The galaxy group optimization (GSO), proposed by Nakarajan et al.^44^ is a new meta-heuristic algorithm for global optimization inspired by galaxy motion. The Fruit Fly Optimization Algorithm (FOA)^45^ is a swarm intelligence method for global optimization based on the foraging behavior of fruit flies. FOA implements an iterative group search for the optimal spatial solution by simulating the foraging process of fruit flies using sharp olfactory and visual predation. The algorithm has been applied to fault diagnosis of analog circuits and wireless sensor networks^46,47^. The Fox optimizer^48^, proposed by Mohammed in the context of hunting behavior exhibited by foxes in the natural world, focuses on foraging actions during prey capture. The Emperor Penguin Optimization Algorithm (EPO)^49^ was proposed by Dhiman et al., inspired by the group behavior of penguins crowded together for heating in winter. Abualigah et al.^12^ proposed a population-based optimization method—Aquila Optimizer (AO), which is based on Aquila’s predation behavior. The optimization process of the AO algorithm corresponds to four hunting behaviors exhibited by Aquila. Vertical soaring with curved high-altitude flight is selected for exploring the search space, while level flight with short glides is employed for divergent exploration within the search space. Slow descent attacks with low-altitude flight are used for convergent exploration within the search space. Finally, diving for walking and grabbing prey corresponds to the algorithm's exploitation phase. The first two behaviors correspond to the exploration phase of the algorithm, while the latter two behaviors correspond to the exploitation phase.

Despite the variations among metaheuristic algorithms, they share a distinct characteristic: the search process is bifurcated into two common phases—exploration and exploitation^52^. The exploration phase entails algorithmically exploring each region of the search space, ensuring that every promising region is thoroughly examined. The strength of the exploration ability is critical in determining whether the algorithm can efficiently locate the globally optimal solution. Meanwhile, the exploitation phase refers to further optimizing the potential areas unearthed during the exploration stage; achieving the right balance between these two stages remains a challenging task^40,53^.

Abualigah et al.^12^ proposed AO as a novel optimization algorithm, inspired by the predation behavior of Aquila. It is characterized by its strong convergence ability and simple implementation when compared to other algorithms. As a result, AO quickly garnered the attention of scholars and has been extensively studied. For instance, Vashishtha et al.^54^ employed the Aquila optimizer to select the ideal filter length adaptively, using autocorrelation energy as their fitness function. Fatani et al.^55^ used the binary version of the Aquila optimizer as a feature selection (FS) technique to dynamically select most relevant features, thereby enhancing classification accuracy. Based on the development version of Aquila optimizer (DAO), Wang et al.^56^ used multi-objective optimization techniques to improve overall product cost and performance efficiency. These applications of AO underscore the algorithm's widespread popularity. In comparison to traditional methods, AO demonstrates superior performance. However, in some cases, due to the lack of population diversity, shortcomings such as slow convergence speed, susceptibility to falling into local optima, and low accuracy may arise. To address challenges arising in the Aquila Optimizer (AO) with the increasing complexity of practical optimization problems, Gao proposed an improved algorithm called the Improved Aquila Optimizer (IAO)^57^. This enhancement aims to specifically address potential shortcomings or a tendency to get stuck in local optima associated with AO. Acknowledging the limitations of the Aquila Optimizer (AO) in dealing with issues such as slow convergence, low convergence accuracy, and susceptibility to local optima in complex optimization problems, Huang introduced a Hybrid Aquila Optimizer (HAO)^58^ based on Gaussian mapping and crossover operators. Zeng presented a Spiral Golden Eagle Optimizer based on Dynamic Gaussian Mutation (SGAO)^50^ to tackle the challenges posed by AO in terms of slow convergence and other complexities. To enhance the late-stage convergence speed of AO, Wang proposed an Enhanced Aquila Optimizer based on a Velocity-Assisted Global Search Mechanism and AO's Adaptive Opposition Learning (VAIAO)^59^. This approach aims to improve the overall performance of Aquila Optimizer. The No Free Lunch (NFL) theorem^60^ logically demonstrates that there cannot be a single meta-heuristic optimization algorithm capable of addressing all optimization problems. This means that while an algorithm may prove to be effective in resolving one optimization problem, it may not work as well when applied to a different one. Therefore, this is motivating continuous development of new meta-heuristic algorithms aimed at resolving various optimization problems.

To address the shortcomings of the Aquila Optimizer (AO), such as slow convergence speed in the later stages of optimization, low optimization accuracy, susceptibility to local optima, and poor performance in high-dimensional and complex problems, this paper introduces a novel optimization algorithm called Chaotic Initialization and New Update Rules Enhanced Aquila Optimization (TEAO). TEAO is specifically designed to overcome the limitations of AO by incorporating chaotic initialization and new update rules. The proposed algorithm makes four key contributions, outlined as follows:

1. Introducing the Tent chaotic map for population initialization ensures an even distribution of the initial solution across the solution space, thereby accelerating the algorithm's convergence speed.
2. To enhance convergence speed while preserving accuracy, we introduce novel exploration and exploitation strategies.
3. TEAO's performance is assessed across 23 benchmark functions and compared with 14 other algorithms.
4. The effectiveness and accuracy of TEAO in solving practical problems are validated through six engineering design problems.

The article is organized as follows: section "Aquila optimization"presents a brief introduction to the AO algorithm, followed by a detailed description of the proposed TEAO algorithm in section "The proposed Aquila optimizer (TEAO)". Section "Experimental results and analysis" outlines the corresponding simulation experiments and provides an in-depth analysis. In section "TEAO is used for the engineering design problems", we apply the proposed TEAO to six classical practical engineering problems. Finally, section "Tension/compression spring design (T/CSD)" summarizes the entire text and offers an outlook for future prospects.

## Aquila optimization

This section primarily introduces the origin of the Aquila Optimization (AO) and the corresponding mathematical model of the algorithm for the algorithm.

### The source of inspiration

Aquila is a popular raptor in the Northern Hemisphere, renowned for its speed, agility, and powerful feet equipped with large, sharp claws. These attributes make it an efficient hunter of ground animals such as rabbits, hares, abyss, groundhogs, squirrels, and more^61^. This bird of prey primarily employs four hunting methods, each with distinct variations. Aquila can swiftly and skillfully switch between hunting methods based on the situation at hand. The first method involves hovering vertically at a high altitude to hunt birds in flight, then ascending rapidly from the ground. Once prey is spotted, Aquila adopts a long, low-angle glide with increasing speed as the wings draw closer^62^. The second and most commonly employed method involves contour flying with a short-range glide attack. Aquila begins the hunt by ascending from a lower level on the ground and can catch both ground animals and birds in flight. The third method involves flying in a straight line at low altitude, gradually descending in the direction of the prey to attack it^63^. The fourth method is Aquila’s ability to walk on land and seize prey with its strong talons^64^.

### The mathematical model of AO

The algorithm is optimized through the simulation of Aquila’s four predatory behaviors, using the following four methods^65^: (1) Vertical high-pitch flight is used to choose the search space; (2) Divergent search space exploration takes place through contour flight and short glide attacks; (3) Convergence search space is utilized by employing low-altitude flight and executing a slow descent attack; (4) Predatory actions are executed through diving, walking, and capturing prey. Each of these strategies will be briefly explained below.

#### Initialization process

Aquila Optimization is a population-based method. The optimization process begins with the population of the candidate solution (${\varvec{X}}$) as depicted in Eq. (1), which is randomly generated between the upper bound (*UB*) and the lower bound (*LB*) constraints of the given problem. The optimal solution obtained so far is provisionally considered as the optimal solution in each iteration.

1 $$\begin{array}{c}X={\left[\begin{array}{ccccc}{x}_{\mathrm{1,1}}& \cdots & {x}_{1,j}& \cdots & {x}_{1,Dim}\\ \vdots & \cdots & \vdots & \cdots & \vdots \\ {x}_{i,1}& \cdots & {x}_{i,j}& \cdots & {x}_{i,Dim}\\ \vdots & \cdots & \vdots & \cdots & \vdots \\ {x}_{N,1}& \cdots & {x}_{N,j}& \cdots & {x}_{N,Dim}\end{array}\right]}\end{array}$$

Here, ${\varvec{X}}$ represents a set of current candidate solutions, which are randomly generated by Eq. (2). ${{\varvec{X}}}_{{\varvec{i}}}$ denotes the decision value (position) of the ${i }$th solution, $N$ is the total number of candidate solutions (population), and $Dim$ represents the size of the problem dimension.

2 $$\begin{array}{c}{{\varvec{X}}}_{{\varvec{i}}{\varvec{j}}}= \, {\text{rand}} \, \times \left(U{B}_{j}-L{B}_{j}\right)+L{B}_{j},i=\mathrm{1,2},\dots ,N,j=\mathrm{1,2},\dots , \, {\text{Dim}}\end{array}$$

Here, $rand$ is a random number, $L{B}_{j}$ is the $j$ ^th^ lower bound of the problem, and $U{B}_{j}$ is the $j$th upper bound of the problem.

#### Step 1: Expanded exploration (X1)

**Figure 1:**
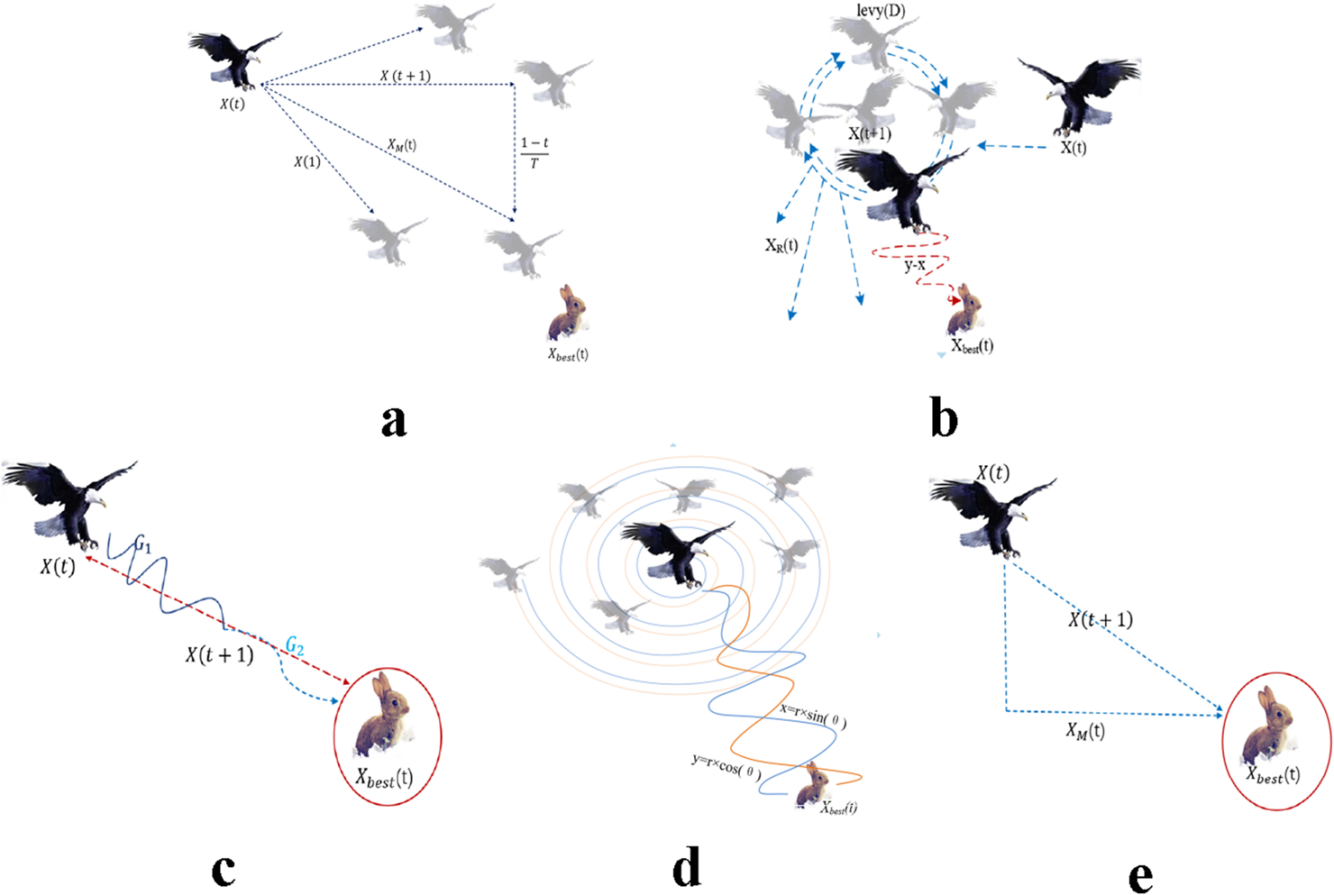
The behavior of the Aquila.

The first method, denoted as ${{\varvec{X}}}_{1}$ in Aquila Optimization, involves identifying the prey area and selecting the best hunting area by soaring to great heights and bending vertically. Aquila’s exploratory abilities are utilized to fly at a significant altitude, determine the search space and prey position. This behavior is depicted in Fig. 1a and is mathematically represented in Eq. (3).

3 $$\begin{array}{c}{{\varvec{X}}}_{1}\left({\varvec{t}}+1\right)={{\varvec{X}}}_{{\varvec{b}}{\varvec{e}}{\varvec{s}}{\varvec{t}}}\left({\varvec{t}}\right)\times \left(1-\frac{t}{T}\right)+\left({{\varvec{X}}}_{{\varvec{M}}}\left({\varvec{t}}\right)-{{\varvec{X}}}_{\text{best }}\left({\varvec{t}}\right)\times \text{ rand }\right) \end{array}$$

Here, ${{\varvec{X}}}_{1}\left({\varvec{t}}+1\right)$ represents the solution of the next iteration generated by the first search method, and ${{\varvec{X}}}_{{\varvec{b}}{\varvec{e}}{\varvec{s}}{\varvec{t}}}\left({\varvec{t}}\right)$ is the optimal solution obtained before the *t*th iteration. The term $\left(1-\frac{t}{T}\right)$ is utilized to regulate the extended search (exploration) based on the number of iterations. ${{\varvec{X}}}_{{\varvec{M}}}\left({\varvec{t}}\right)$ denotes the average position of the current solution at the *t*th iteration, calculated using Eq. (4). And $rand$ is a random number between 0 and 1, where $t$ and $T$ represent the current iteration and the maximum number of iterations, respectively.

4 $$\begin{array}{c}{{\varvec{X}}}_{{\varvec{M}}}\left({\varvec{t}}\right)=\frac{1}{N}\sum_{i=1}^{N} {{\varvec{X}}}_{{\varvec{i}}}\left({\varvec{t}}\right),\forall j=\mathrm{1,2},\dots ,Dim\end{array}$$

#### Step 2: Narrowed exploration (*X*_*2*_)

The second method in Aquila Optimization involves hovering over the target prey after identifying the prey area from a great height. This technique, known as contour flight and short glide attack, enables Aquila to thoroughly explore the target prey area before executing a short glide attack. Figure 1-b illustrates Aquila’s behavior during contour flight and short glide attack. Mathematically, this behavior can be expressed as Eq. (5).

5 $$\begin{array}{c}{{\varvec{X}}}_{2}(t+1)={{\varvec{X}}}_{\text{best }}(t)\times Levy(D)+{{\varvec{X}}}_{{\varvec{R}}}(t)+(y-x{)}^{ }\times rand\end{array}$$

where ${{\varvec{X}}}_{2}({\varvec{t}}\boldsymbol{ }+\boldsymbol{ }1)$ represents the next iterative solution at time $t$*,* generated by the second search method. $Levy(D)$ is the $Levy$ flight distribution function, calculated using Eq. (6), and ${{\varvec{X}}}_{{\varvec{R}}}({\varvec{t}})$ is a random solution in the range $[1,N]$ at the *i*th iteration.

6 $$\begin{array}{c}\mathit{Levy}\left(D\right)=s\times \frac{u\times \sigma }{|v{|}^{\frac{1}{\beta }}}\end{array}$$

Here, $s$ is a constant fixed at 0.01, $u$ and $v$ are random numbers between 0 and 1. The value of $\sigma$ is calculated using Eq. (7).

7 $$\begin{array}{c}\sigma =\left(\frac{\Gamma \left(1+\beta \right)\times {\text{sine}}\left(\frac{\pi \times \beta }{2}\right)}{\Gamma \left(\frac{1+\beta }{2}\right)\times \beta \times 2\left(\frac{\beta -1}{2}\right)}\right)\end{array}$$

Here, $\beta$ is a constant value fixed at 1.5. In Eq. (5), *y* and *x* are used to represent the spiral shape in the search, The calculation is as follows:

8 $$\begin{array}{c}x=r\times {\text{cos}}\left(\theta \right)\end{array}$$

9 $$\begin{array}{c}y=r\times {\text{sin}}\left(\theta \right)\end{array}$$

10 $$\begin{array}{c} r={r}_{1}+U\times {D}_{1}\end{array}$$

11 $$\begin{array}{c}\theta =-\omega \times {D}_{1}+{\theta }_{1}\end{array}$$

12 $$\begin{array}{c}{\theta }_{1}=\frac{3\times \pi }{2}\end{array}$$

The value of ${r}_{1}$ between 1 and 20, serves as the fixed number of search cycles. $U$ is a small value fixed at 0.00565, ${D}_{1}$ is an integer ranging from 1 to the search space length $Dim$, and $\omega$ is a small value fixed at 0.005, Fig. 1c illustrates the spiral behavior of the Aquila.

#### Step 3: Expanded exploitation (*X*_*3*_)

The third method in Aquila Optimization involves accurately specifying the prey area and preparing to land and attack. Aquila descends vertically and carries out a preliminary attack to gauge the response of the prey. This method is commonly known as low the altitude slow drop attack. Aquila uses the selected area of the target to approach the prey and execute an attack. Figure 1-d illustrates Aquila’s slow descent attack behavior, which can be mathematically expressed as Eq. (13).

13 $$\begin{array}{c}{{\varvec{X}}}_{3}\left({\varvec{t}}+1\right)=\left({{\varvec{X}}}_{\text{best }}\left({\varvec{t}}\right)-{{\varvec{X}}}_{{\varvec{M}}}\left({\varvec{t}}\right)\right)\times \alpha - \, {\text{rand}} \, +\left(\left(UB-LB\right)\times \text{ rand} \, +LB\right)\times \delta \end{array}$$

Here,${{\varvec{X}}}_{3}({\varvec{t}}\boldsymbol{ }+\boldsymbol{ }1)$ represents the solution of the next iteration at time *t*, generated by the third search method. The parameters $\alpha$ and $\delta$ are mining adjustment parameters determined in this paper, set to smaller values (0.1).

#### Step 4: Narrowed exploitation (*X*_*4*_)

The fourth method in Aquila Optimization involves attacking the prey on land using random motion after Aquila has approached the target. This method is called walking and catching prey, with the final attack on the prey being carried out in the last position. Aquila’s walking and preying behavior is illustrated in Fig. 1-e and can be mathematically represented by Eq. (14).

14 $$\begin{array}{c}{{\varvec{X}}}_{4}\left({\varvec{t}}+1\right)= QF\times {{\varvec{X}}}_{\text{best }}\left({\varvec{t}}\right)-\left({{\varvec{G}}}_{1}\times {\varvec{X}}\left({\varvec{t}}\right)\times \, {\text{rand}} \, \right)-{{\varvec{G}}}_{2}\times {\varvec{Levy}}\left({\varvec{D}}\right)+ \, {\text{rand}}\times {{\varvec{G}}}_{1}\end{array}$$

Here,${{\varvec{X}}}_{4}({\varvec{t}}\boldsymbol{ }+\boldsymbol{ }1)$ refers to the solution generated by the fourth search method for the next iteration at time $t$. The quality function used to balance the search strategy is *QF*, calculated using Eq. (15).${ G}_{1}$ represents the diverse movements of Aquila used to track the prey while escaping and is generated by Eq. (16).${G}_{2}$ is a decreasing value ranging from 2 to 0, indicating the flight slope of Aquila used to pursue the prey as it escapes from the first position to the last position. Equation (17) is employed to generate ${G}_{2}$. $X(t )$ represents the current solution at the *t*th iteration.

15 $$\begin{array}{c}QF\left(t\right)={t}^{\frac{2\times rand-1}{(1-T{)}^{2}}}\end{array}$$

16 $$\begin{array}{c}{G}_{1}=2\times \, {\text{rand}} \, -1 \end{array}$$

17 $$\begin{array}{c}{G}_{2}=2\times \left(1-\frac{t}{T}\right)\end{array}$$

**Figure 2:**
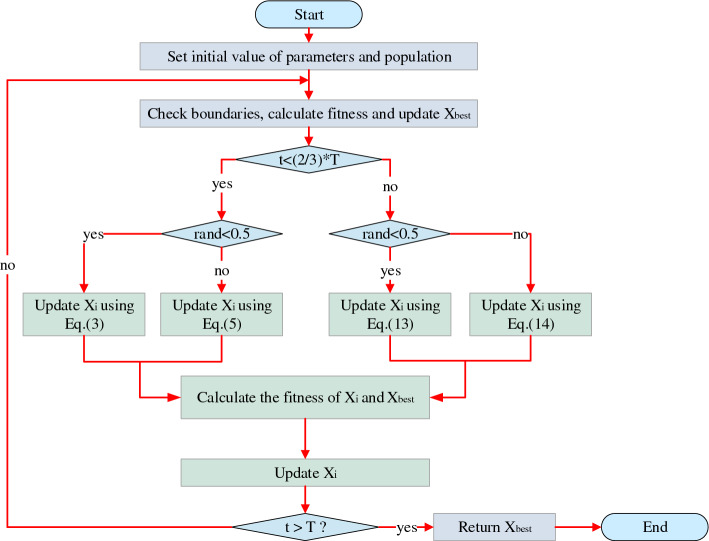
Flow chart of AO.

In summary, AO flow chart Fig. 2 shows.

## The proposed Aquila optimizer (TEAO)

This section introduces the proposed TEAO algorithm, designed to tackle the challenges of slow convergence speed, low precision, and vulnerability to local optima commonly associated with AO. To enhance the performance of AO, we have introduced several modifications to the algorithm, elaborated in detail in the following sections.

### Population initialization strategy

Despite the uneven distribution and pronounced randomness in the initial Aquila population, AO still encounters two major challenges—premature convergence and susceptibility to local optima. Chaos, known for its randomness, ergodicity, and sensitivity to initial conditions, holds the potential for expediting convergence in algorithms. Compared to other chaotic mappings, such as the Logistic chaotic mapping, the Tent chaotic mapping exhibits a more uniform traversal of the state space. This suggests that, during the chaotic mapping process, the state variables can thoroughly explore the state space, leading to richer chaotic behaviors. Moreover, owing to the Tent chaotic mapping's faster search speed, it can accelerate the identification of optimal solutions in optimization problems. Therefore, this paper proposes the use of the Tent mapping to generate chaotic sequences^51–53^ for the initialization of the Aquila population, with the aim of achieving a more uniform distribution of initial solutions across the solution space. The Tent chaotic initialization population can be mathematically represented by Eq. (18).

18 $$\begin{array}{c}{{\varvec{X}}}_{{\varvec{i}}{\varvec{j}}}={Z}_{i}\times \left(U{B}_{j}-L{B}_{j}\right)+L{B}_{j},i=\mathrm{1,2},\dots ,N,j=\mathrm{1,2},\dots ,Dim\end{array}$$

Here, ${Z}_{i}$ is chaotic sequences.

19 $$\begin{array}{c}{Z}_{i+1}=\left\{\begin{array}{c}\frac{{Z}_{i}}{u} ,0\le {Z}_{i}\le u,\\ \frac{1-{Z}_{i}}{1-u} ,u<{Z}_{i}\le 1\end{array}\right.\end{array}$$

**Figure 3:**
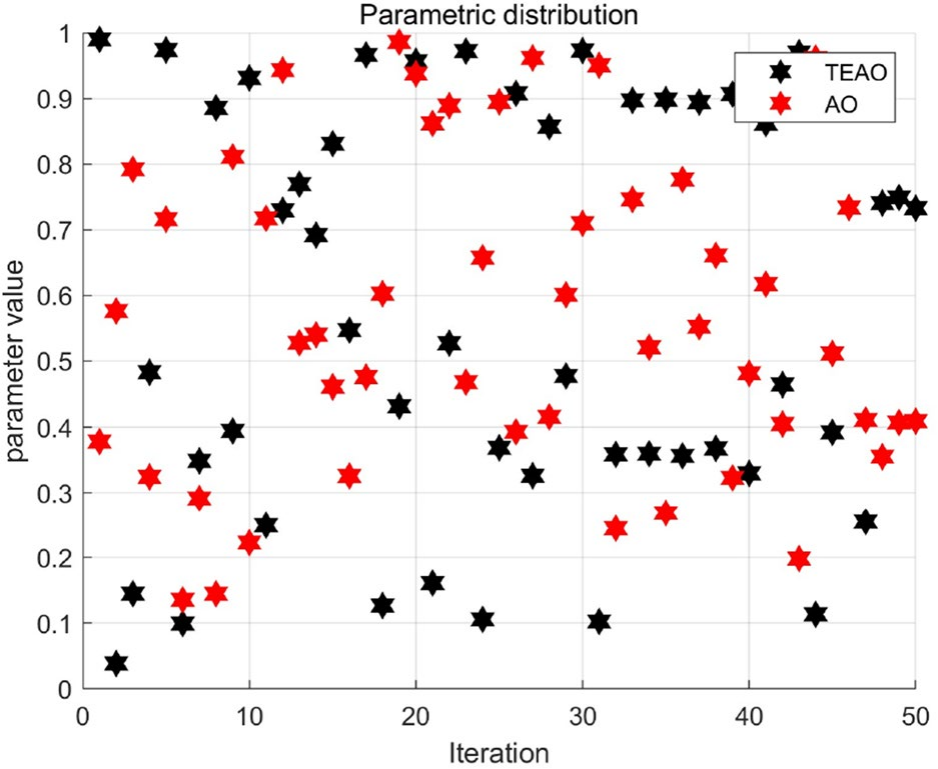
Comparison of population distribution between TEAO and AO.

Here, ${Z}_{i}$ represents the current iterative chaotic sequence, ${Z}_{i+1}$ is the chaotic sequence of the next iteration, with $u\subset rand(\mathrm{0,1})$. Compared to random-based initialization, this chaos-based initialization method aims to distribute the population in the solution space as widely as possible. Figure 3 illustrates the distribution of random numbers generated by different initialization methods. The population distribution of TEAO is broader, enabling TEAO to explore a wider search range when hunting for prey in Aquila. As a result, TEAO enhances the algorithm's global search capability, contributing to an improvement in the convergence speed.

### The new position update rules

This section outlines the proposed approach for updating Aquila's position during the four hunting methods. The introduction of new position update rules significantly improves the algorithm's exploration and exploitation capabilities, resulting in accelerated convergence speed and enhanced algorithmic accuracy. These improvements enable the algorithm to more effectively address optimization problems.

Expanded exploration $${({\varvec{X}}}_{1})$$

**Figure 4:**
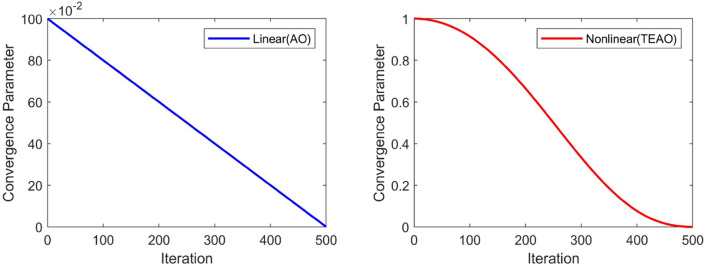
Convergence factor comparison diagram.

To accelerate the convergence speed and improve the convergence accuracy of the algorithm, the convergence factor has been modified to ${\left(1-\frac{t}{T}\right)}^{\left(\frac{2\times t}{T}\right)}$, where t represents the current iteration, and T is the total number of iterations. The comparison between the values of the improved convergence factor and the original convergence factor is shown in Fig. 4. It can be observed that the curve of the novel nonlinear convergence strategy remains relatively flat during the early phase of the iteration, indicating that the search agent is extensively exploring the entire search space. As the iteration progresses, the curve begins to decline rapidly, signifying faster convergence of the algorithm. In contrast, the linear convergence curve focuses more on exploitation, which can lead to premature convergence. Our proposed approach for updating the position of Aquila during its first hunting method can be mathematically expressed as Eq. (20).

20 $$\begin{array}{c}{{\varvec{X}}}_{1}\left({\varvec{t}}+1\right)={{\varvec{X}}}_{{\text{b}}\text{est }}\left({\varvec{t}}\right)\times {\left(1-\frac{t}{T}\right)}^{\left(\frac{2\times t}{T}\right)}+ \left({{\varvec{X}}}_{{\varvec{M}}}\left({\varvec{t}}\right)-{{\varvec{X}}}_{\text{best }}\left({\varvec{t}}\right)\right)\times \, {\text{rand}}\end{array}$$

Narrowed exploration $${({\varvec{X}}}_{2})$$

We have introduced a new position update rule for Aquila during its second hunting method, expressed mathematically as Eq. (21).

21 $$\begin{array}{c}{{\varvec{X}}}_{2}\left({\varvec{t}}+1\right)={{\varvec{X}}}_{\text{best }}\left({\varvec{t}}\right)+ \left({{\varvec{X}}}_{{\varvec{A}}1}\left({\varvec{t}}\right)-{{\varvec{X}}}_{{\varvec{A}}2}\left({\varvec{t}}\right)\right)\times \, {\text{rand}}\end{array}$$

Here, ${{\varvec{X}}}_{{\varvec{A}}1}\left({\varvec{t}}\right)$ and ${{\varvec{X}}}_{{\varvec{A}}2}\left({\varvec{t}}\right)$ represent the random candidate solutions of the ${t}^{th}$ iteration.

Expanded exploitation $${({\varvec{X}}}_{3})$$

The approach we propose for updating Aquila's position during its third hunting method can be mathematically expressed as Eq. (22).

22 $${{\varvec{X}}}_{3}\left({\varvec{t}}+1\right)={{\varvec{X}}}_({\varvec{t}})-\left({{\varvec{X}}}_({\varvec{t}})-{{\varvec{X}}}_{{\varvec{b}}{\varvec{e}}{\varvec{s}}{\varvec{t}}}\left({\varvec{t}}\right)\times rand\right)\times {\left(1-\frac{t}{T}\right)}^{\left(\frac{2\times t}{T}\right)}\times phi \times rand$$

Here, ${{\varvec{X}}}\left({\varvec{t}}\right)$ represents the current iteration position, and $phi$ is a random number so that the elements are between $\left(-\mathrm{1,1}\right)$.

Narrowed exploitation (***X***_***4***_)

We have introduced a new position update rule for Aquila in its fourth hunting method, mathematically expressed as Eq. (23).

23 $$\begin{array}{c}{{\varvec{X}}}_{4}\left({\varvec{t}}+1\right)={{\varvec{X}}}_{\text{best }}\left({\varvec{t}}\right)\times \mathit{Levy}\left(Dim\right)\end{array}$$

**Figure 5:**
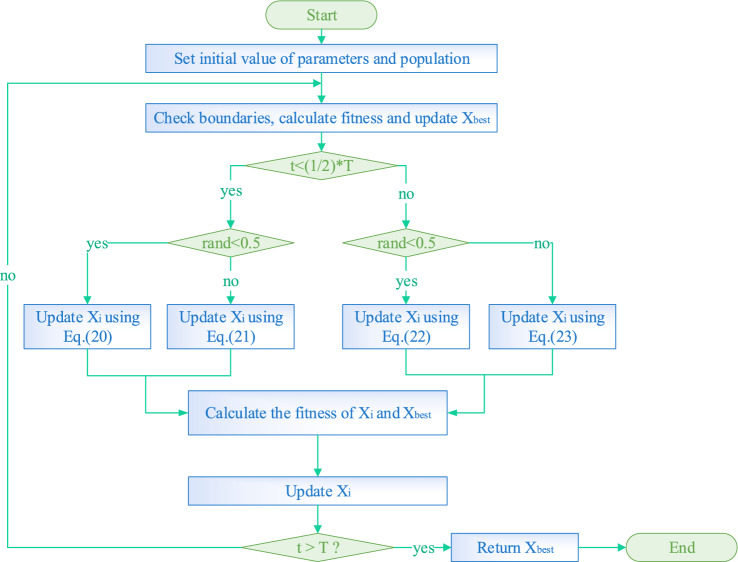
Flow chart of TEAO.

In summary, the pseudo-code for the TEAO algorithm is available in Table 1, and the flow chart is illustrated in Fig. 5.

**Table 1:**
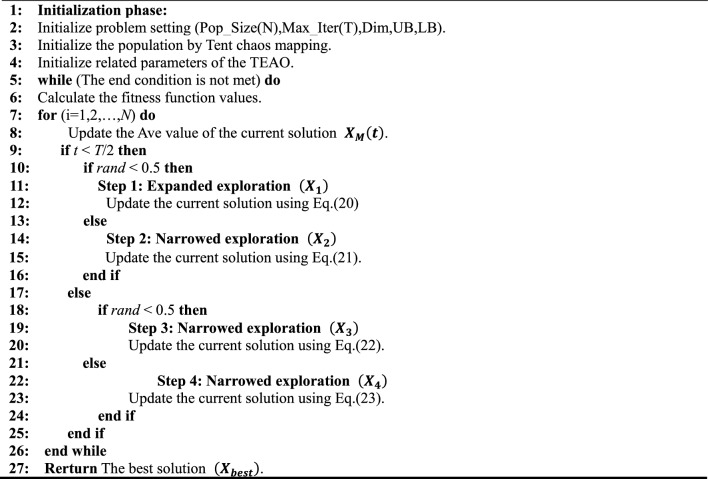
Pseudo-code of TEAO algorithm.

### Time complexity analysis

The runtime of algorithms can vary for the same optimization problem. One effective way to assess an algorithm’s runtime is through its computational complexity^34^. This paper employs big O complexity notation to analyze and compare the time complexity of AO and TEAO algorithms^66,67^. The computational complexity of solving the problem is *O*(*N*), while the initialization process is *O*(*T* × *N*). This process involves finding the optimal position and updating the position of all solutions, where *T* represents the total iterations and *Dim* refers to the dimension of the problem. Therefore, the computational complexity of AO is *O*(*N* × (*T* × *D* + *1*)). TEAO has the same complexity as it does not introduce any new cycles, resulting in a time complexity of *O*(*N* × (*T* × *D* + *1*)).

## Experimental results and analysis

In this section, we assess the optimization performance of TEAO across 23 classical benchmark functions 75 and compare it with other 14 popular optimization algorithms. The algorithms are executed using a consistent system configuration, implemented on a desktop computer featuring a 13th Intel(R) Core (TM) i5-13400 (16 CPUs), ~ 2.5 GHz processor and 16 GB RAM. These experiments were conducted using the MATLAB 2022b platform. The experimental results and statistical analysis are presented in the following sections.

### The 23 classical benchmark functions

To assess the exploration and exploitation capabilities of TEAO in the search space, we conducted experiments using a set of 23 classic benchmark functions, as detailed in references^68,69^. This set comprises 7 unimodal test functions (F1–F7) outlined in Table 2, used to evaluate the exploitation performance of TEAO. Unimodal functions have only one optimal value and no local optimal values. In contrast, Table 3 provides six multimodal test functions (F8–F13) containing multiple local optimal values, enabling us to evaluate whether the algorithm can identify the global optimal solution and avoid getting stuck in local optima. Finally, Table 4 presents the fixed-dimensional multimodal test functions (F14–F23), used to assess the exploration performance of the algorithm in the context of complex, low-dimensional test functions.

**Table 2:** Unimodal benchmark functions.

Function	Dimensions	Range	*f*_*min*_
${f}_{1}(x)=\sum_{i=1}^{n} {x}_{i}^{2}$	30/100/500/1000	[− 100, 100]	0
${f}_{2}(x)=\sum_{i=1}^{n} \left|{x}_{i}\right|+\prod_{i=1}^{n} \left|{x}_{i}\right|$	30/100/500/1000	[− 10, 10]	0
${f}_{3}(x)=\sum_{i=1}^{n} {\left(\sum_{j-1}^{i} {x}_{j}\right)}^{2}$	30/100/500/1000	[− 100, 100]	0
${f}_{4}(x)= {max}_{i}\left\{\left|{x}_{i}\right|,1\leq i\leq n\right\}$	30/100/500/1000	[− 100, 100]	0
${f}_{5}(x)=\sum_{i=1}^{n-1} \left[100{\left({x}_{i+1}-{x}_{i}^{2}\right)}^{2}+{\left({x}_{i}-1\right)}^{2}\right]$	30/100/500/1000	[− 30, 30]	0
${f}_{6}(x)=\sum_{i=1}^{n} {\left(\left[{x}_{i}+0.5\right]\right)}^{2}$	30/100/500/1000	[− 100, 100]	0
${f}_{7}(x)=\sum_{i=1}^{n} i{x}_{i}^{4}+{\text{random}}[\mathrm{0,1})$	30/100/500/1000	[− 1.28, − 1.28]	0

**Table 3:** Multimodal benchmark functions.

Function	Dimensions	Range	*f*_*min*_
${F}_{8}(x)=\sum_{i=1}^{n} -{x}_{i}{\text{sin}}\left(\sqrt{\left|{x}_{i}\right|}\right)$	30/100/500/1000	[− 500, 500]	− 418.9829 × d
${F}_{9}(x)=\sum_{i=1}^{n} \left[{x}_{i}^{2}-10{\text{cos}}\left(2\pi {x}_{i}\right)+10\right]$	30/100/500/1000	[5.12, 5.12]	0
${F}_{10}(x)=-20{\text{exp}}\left(-0.2\sqrt{\frac{1}{n}\sum_{i=1}^{n} {x}_{i}^{2}}\right)-{\text{exp}}\left(\frac{1}{n}\sum_{i=1}^{n} {\text{cos}}\left(2\pi {x}_{i}\right)\right)+20+e$	30/100/500/1000	[− 32, 32]	0
${F}_{11}(x)=\frac{1}{4000}\sum_{i=1}^{n} {x}_{i}^{2}-\prod_{i=1}^{n} {\text{cos}}\left(\frac{{x}_{i}}{\sqrt{i}}\right)+1$	30/100/500/1000	[600, 600]	0
${F}_{12}(x)=\frac{\pi }{n}\left\{10{\text{sin}}\left(\pi {y}_{1}\right)+\sum_{i=1}^{n-1} {\left({y}_{i}-1\right)}^{2}\left[1+10{{\text{sin}}}^{2}\left(\pi {y}_{i+1}\right)\right]+{\left({y}_{n}-1\right)}^{2}\right\}+\sum_{i=1}^{n} u\left({x}_{i},\mathrm{10,100,4}\right)$	30/100/500/1000	[− 50, 50]	0
$${F}_{13}\left(x\right)=0.1\left\{\begin{array}{c}{{\text{sin}}}^{2}\left(3\pi {x}_{1}\right)+\sum_{i=1}^{n} {\left({x}_{i}-1\right)}^{2}\left[1+{{\text{sin}}}^{2}\left(3\pi {x}_{i}+1\right)\right]\\ +{\left({x}_{n}-1\right)}^{2}\left[1+{{\text{sin}}}^{2}\left(2\pi {x}_{n}\right)\right]\end{array}\right\}+\sum_{i=1}^{n} u\left({x}_{i},\mathrm{5,100,4}\right)$$	30/100/500/1000	[− 50, 50]	0

**Table 4:** Fixed-dimension multimodal benchmark functions.

Function	Dimensions	Range	*f*_*min*_
${F}_{14}(x)={\left(\frac{1}{500}+\sum_{j=1}^{25} \frac{1}{j+\sum_{i=1}^{2} {\left({x}_{i}-{a}_{ij}\right)}^{6}}\right)}^{-1}$	2	[65.536, 65.536]	0.998
${F}_{15}(x)=\sum_{i=1}^{11} {\left[{a}_{i}-\frac{{x}_{1}\left({b}_{i}^{2}+{b}_{i}{x}_{2}\right)}{{b}_{i}^{2}+{b}_{i}{x}_{3}+{x}_{4}}\right]}^{2}$	4	[− 5, 5]	0.0003
${F}_{16}(x)=4{x}_{1}^{2}-2\cdot 1{x}_{1}^{4}+\frac{1}{3}{x}_{1}^{6}+{x}_{1}{x}_{2}-4{x}_{2}^{2}+4{x}_{2}^{4}$	2	[− 5, 5]	− 1.0316
${F}_{17}(x)={\left({x}_{2}-\frac{5.1}{4{\pi }^{2}}{x}_{1}^{2}+\frac{5}{\pi }{x}_{1}-6\right)}^{2}+10\left(1-\frac{1}{8\pi }\right){\text{cos}}{x}_{1}+10$	2	[− 5, 5]	0.398
${F}_{18}\left(x\right)=\left[1+{\left({x}_{1}+{x}_{2}+1\right)}^{2}\left(19-14{x}_{1}+3{x}_{1}^{2}-14{x}_{2}+6{x}_{1}{x}_{2}+3{x}_{2}^{2}\right)\right]$ $\times \left[30+{\left(2{x}_{1}-3{x}_{2}\right)}^{2}\times \left(18-32{x}_{1}+12{x}_{1}^{2}+48{x}_{2}-36{x}_{1}{x}_{2}+27{x}_{2}^{2}\right)\right]$	2	[− 2, 2]	3
${F}_{19}(x)=-\sum_{i=1}^{4} {c}_{i}{\text{exp}}\left(-\sum_{j=1}^{3} {a}_{ij}{\left({x}_{j}-{p}_{ij}\right)}^{2}\right)$	3	[0, 1]	− 3.86
${F}_{20}(x)=-\sum_{i=1}^{4} {c}_{i}{\text{exp}}\left(-\sum_{j=1}^{6} {a}_{ij}{\left({x}_{j}-{p}_{ij}\right)}^{2}\right)$	6	[0, 1]	− 3.32
${F}_{21}(x)=-\sum_{i=1}^{5} {\left[\left(X-{a}_{i}\right){\left(X-{a}_{i}\right)}^{T}+{c}_{i}\right]}^{-1}$	4	[0, 10]	− 10.1532
${F}_{22}(x)=-\sum_{i=1}^{7} {\left[\left(X-{a}_{i}\right){\left(X-{a}_{i}\right)}^{T}+{c}_{i}\right]}^{-1}$	4	[0, 10]	− 10.4028
${F}_{23}(x)=-\sum_{i=1}^{10} {\left[\left(X-{a}_{i}\right){\left(X-{a}_{i}\right)}^{T}+{c}_{i}\right]}^{-1}$	4	[0, 10]	− 10.5364

### Qualitative analysis

In this section, we employ the set of 23 classical benchmark functions to validate TEAO in terms of exploration and exploitation balance, as well as convergence behavior in 30 dimensions.

#### Exploration and exploitation

Exploration and exploitation are two crucial factors among metaheuristic algorithms. Exploration involves searching for new solutions in the solution space, aiming to discover better solutions in unknown regions. On the other hand, exploitation focuses on known solution spaces and conducts searches within the local neighborhoods of solutions to find potentially superior solutions. A well-balanced combination of exploration and exploitation not only helps the algorithm converge quickly to optimal solutions, enhancing search efficiency, but also allows for flexibility in addressing diverse optimization problems and complexities, showcasing exceptional adaptability and robustness. A high-quality algorithm should strike a good balance between these two factors. Therefore, we use Eqs. (24) and (25) to calculate the percentages of exploration and exploitation, respectively, allowing us to assess the algorithm's balance between these two factors. $Div(t)$ is a measure of dimension diversity calculated by Eq. (26). Here, ${x}_{id}$ represents the position of the *i*th dimension, and ${Div}_{max}$ denotes the maximum diversity throughout the entire iteration process.

24 $$\begin{array}{c}Exploration\left(\%\right)=\frac{Div\left(t\right)}{{Div}_{max}}\times 100\end{array}$$

25 $$\begin{array}{c}Exploitation\left(\%\right)=\frac{\left|Div\left(t\right)-{Div}_{max}\right|}{{Div}_{max}}\times 100\end{array}$$

26 $$\begin{array}{c}Div\left(t\right)=\frac{1}{D}\sum_{d=1}^{D}\frac{1}{N}\sum_{i=1}^{N}\left|median\left({x}_{d}\left(t\right)\right)-{x}_{id}\left(t\right)\right|\end{array}$$

**Figure 6:**
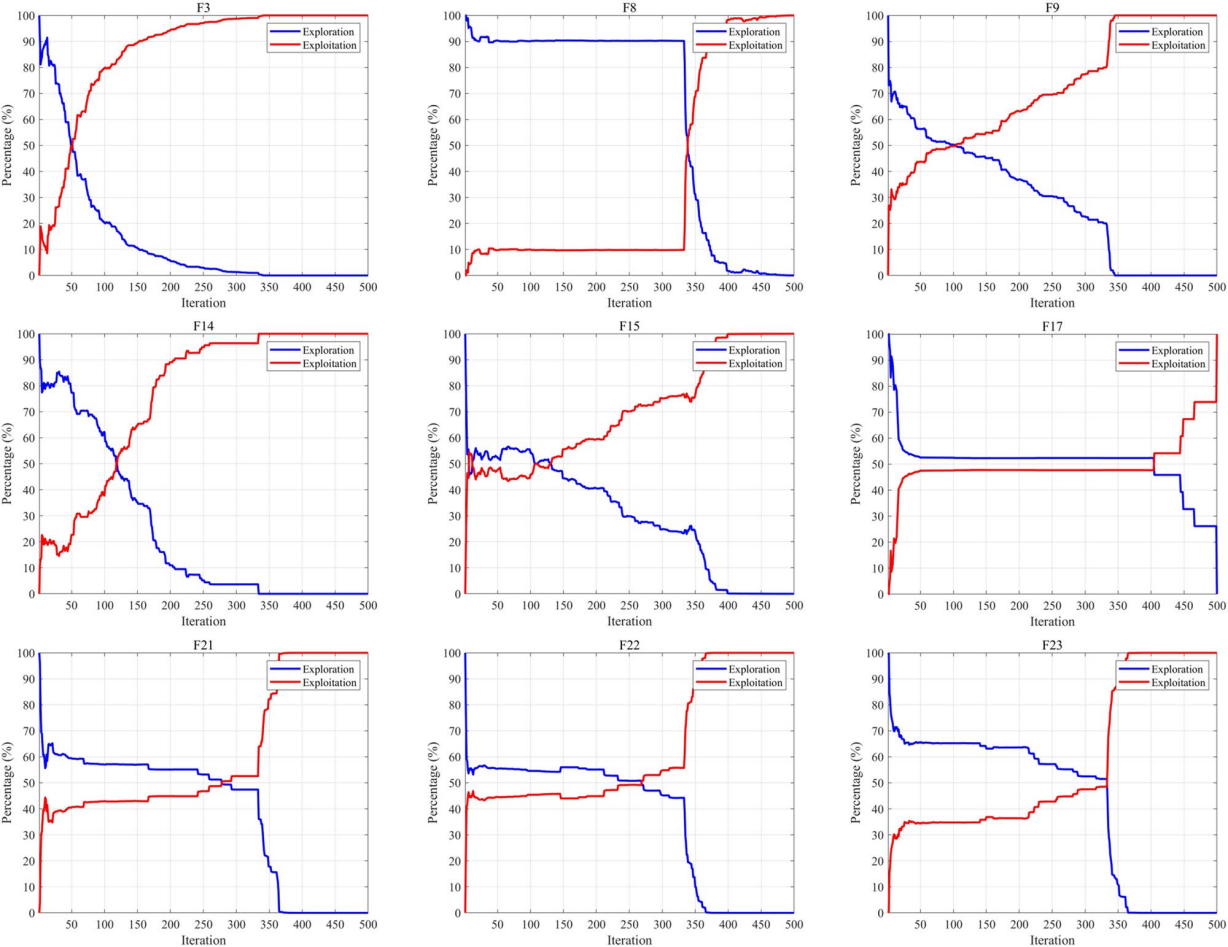
Balance between exploration and exploitation.

Figure 6 intuitively illustrates the balance between exploration and exploitation in TEAO using the 30-dimensional classical benchmark functions. From the graph, it is evident that the intersection point of the exploration and exploitation ratio in the TEAO algorithm primarily occurs during the mid-iterations of the problem search process. In the initial stages, there is a comprehensive exploration of the global search space, gradually transitioning into the phase of local exploitation. It's worth noting that the TEAO algorithm maintains a relatively high exploitation ratio in the later iterations across all functions, contributing to enhanced problem convergence speed and search precision. The TEAO algorithm maintains a dynamic equilibrium between exploration and exploitation throughout the iteration process. Therefore, TEAO exhibits outstanding advantages in avoiding local optima and premature convergence.

#### Convergence behavior analysis

**Figure 7:**
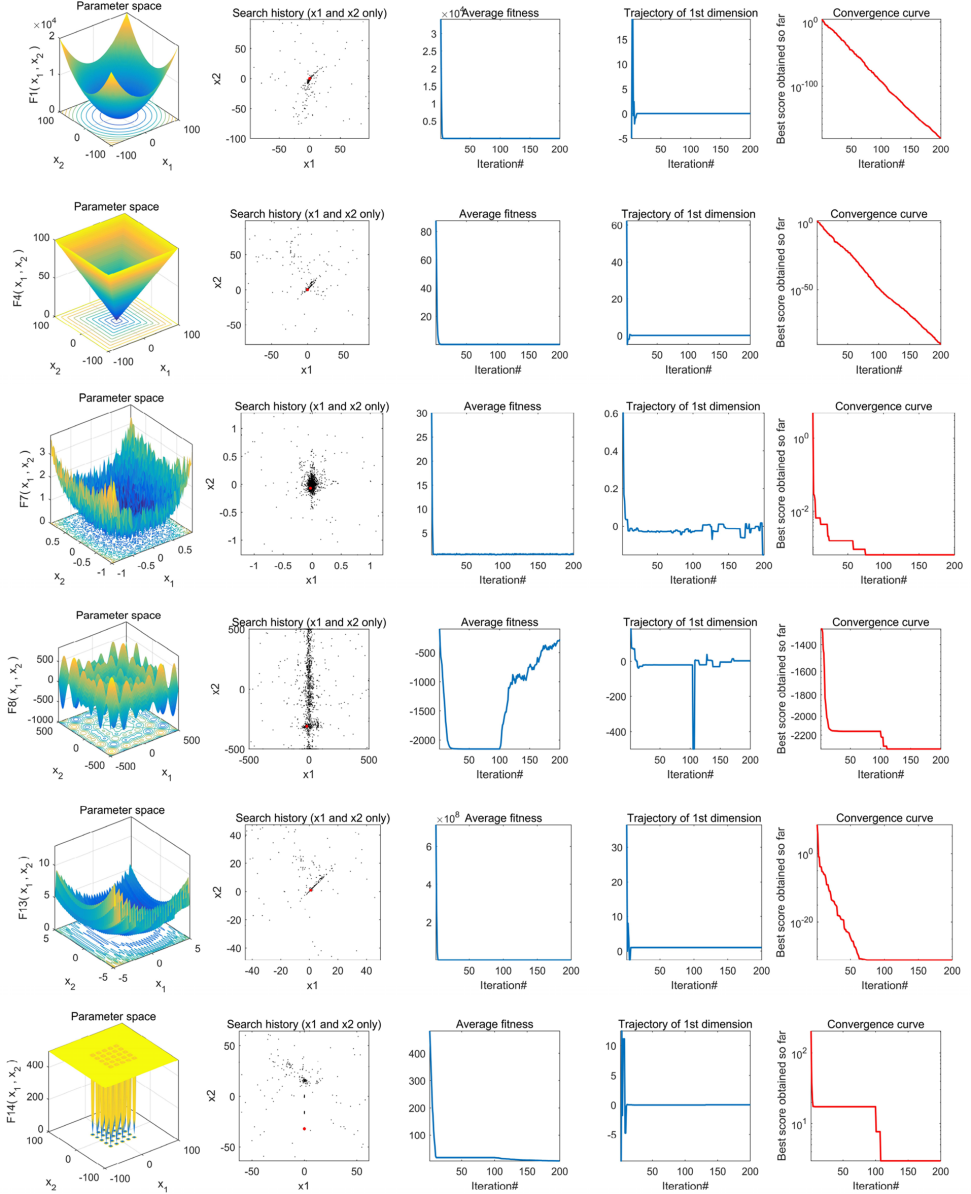

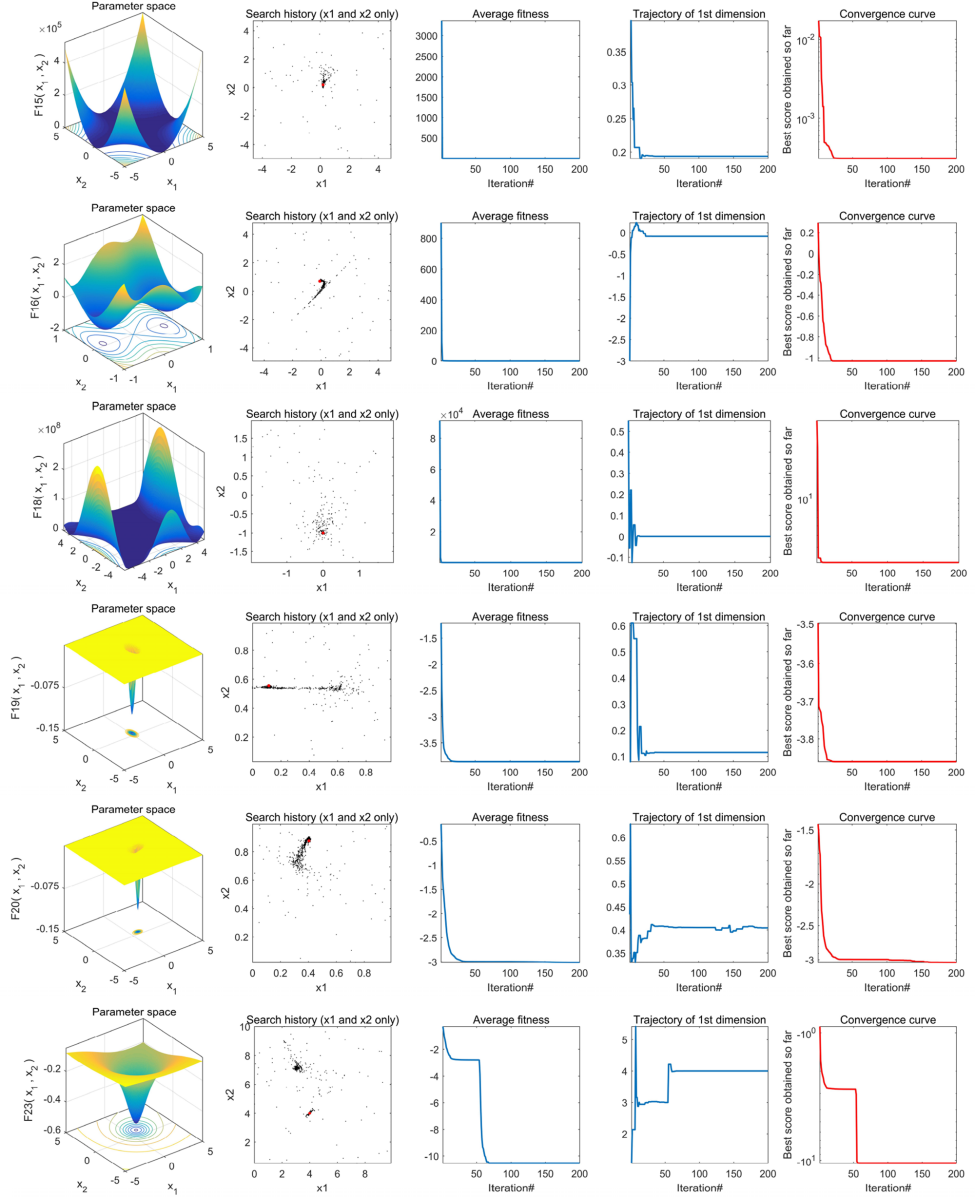
Convergence behaviors of TEAO in the search process.

To conduct a more thorough examination of TEAO's performance, we generated the convergence behavior curves for TEAO, with 200 iterations and a fixed population size of 50. The experimental findings are portrayed in Fig. 7, presenting a total of 12 benchmark function images. Specifically, the first column displays the two-dimensional diagrams of the benchmark functions, while the second column shows the distribution of Aquila throughout the search process. Based on these results, it is clearly that Aquila is uniformly spread across the search space, indicating that the algorithm can effectively explore the search space without becoming trapped in local optima. Additionally, Aquila's position is closely aligns with the optimal solution, indicative of TEAO's strong exploration and exploitation performance.

In addition, the third column represents the average fitness change of the solution during the overall iteration process. The average fitness value is higher at the beginning of the iteration, but after 20 iterations, the average fitness value is smaller and tends to stabilize, indicating that TEAO only needs a small number of iterations to converge to the optimal solution. The fourth column illustrates the trajectory of the best search agent in the first dimension. It can be observed from the graph that the position of Aquila undergoes significant changes at the initial stage of iteration, aiding Aquila in continuous exploration and exploitation of new areas, which is beneficial for finding global optimization and avoiding local optima. The last column shows the convergence curve of TEAO. In the unimodal test function, the convergence curve of the algorithm is relatively smooth. This is because the unimodal test function lacks local optimal values and can easily converge to the optimal solution. In contrast, the multimodal test function has many local optimal solutions, requiring TEAO to constantly escape local optima and search for the global optimum in the iterative process, resulting in a mostly stepped convergence curve. Overall, from these four evaluation indicators, it is evident that our proposed TEAO demonstrates strong convergence performance.

### Quantitative analysis

In this section, we compare TEAO with 14 other algorithms across the 23 classical benchmark functions to assess its performance.

#### Competing algorithms and parameter settings

We compare the TEAO results with 14 other popular optimization algorithms, including the original Aquila Optimizer (AO)^12^, Particle Swarm Optimization (PSO)^15^, Grey Wolf Optimizer (GWO)^31^, Harris Hawks Optimizer (HHO)^70^, Whale Optimization Algorithm (WOA)^71^, Sparrow Search Algorithm (SSA)^72^, Constriction Coefficient-Based Particle Swarm Optimization and Gravitational Search Algorithm (CPSOGSA)^73^, African Vultures Optimization Algorithm (AVOA)^74^, Snake Optimizer (SO)^75^, Runge Kutta Method (RUN)^76^, Golden jackal optimization (GJO)^77^, Fox Optimizer (FOX)^48^, Learner Performance Based Behavior Algorithm (LPB)^21^ and Dung Beetle Optimizer (DBO)^78^. Table 5 provides the original parameter settings for these algorithms. During the experiment, we set the search agents of all algorithms to 50, the maximum number of iterations to 500, and each algorithm to run independently 30 times. The best results for each test function and its corresponding dimension are highlighted in bold.

**Table 5:** Parameter settings for the comparative algorithms.

Algorithm	Parameter	Value
GJO	E1, E0	[0,1.5], [− 1, 2]
GWO	a r1, r2	Linearly decreased from 2 to 0 random
AO	U, ω, α, δ G1, G2	0.00565, 0.005, 0.1, 0.1 Linearly decreased from 2 to 0
	p	0.5
PSO	c1, c2 w	2 W_max_ = 0.9, W_min_ = 0.2
SSA	c1	Decreased from 2 to 0
AVOA	L1, L2, w, p1, p2, p3 α, β, γ	0.8, 0.2, 2.5, 0.6, 0.4, 0.6 0.8,0.2,2.5
WOA	a1	Linearly decreased from 2 to 0
	a2	Linearly increased from − 1 to -2
CPSOGSA	φ1, φ2	2.05, 2.05
HHO	e	It varies randomly in the range (-2,2)
RUN	g, β r	the random number in [0,2] an integer number 1, 0, -1
SO	c1, c2, c3	0.5, 0.05, 2
LPB	Pc, Pm, gamma, mu	0.6, 0.3, 0.05, 0.03
FOX	c1, c2	0.5, 0.82
DBO	P_percent	0.2

#### Experimental analysis of different dimensions of TEAO

In this section, for a better evaluation of TEAO's performance, we conducted comparative validations using the 23 classical benchmark functions. These functions can be categorized into three groups: unimodal benchmark functions, multimodal benchmark functions, and fixed-dimension multimodal benchmark functions. Unimodal benchmark functions possess only one global optimum, without any local optima, making them suitable for assessing the development performance of the algorithm. On the other hand, multimodal benchmark functions feature multiple local optima, primarily serving to evaluate the algorithm's ability to discover the global optimum and escape local optima. The latter categories of functions are employed to assess the algorithm's capability in handling complex continuous problems.

**Figure 8:**
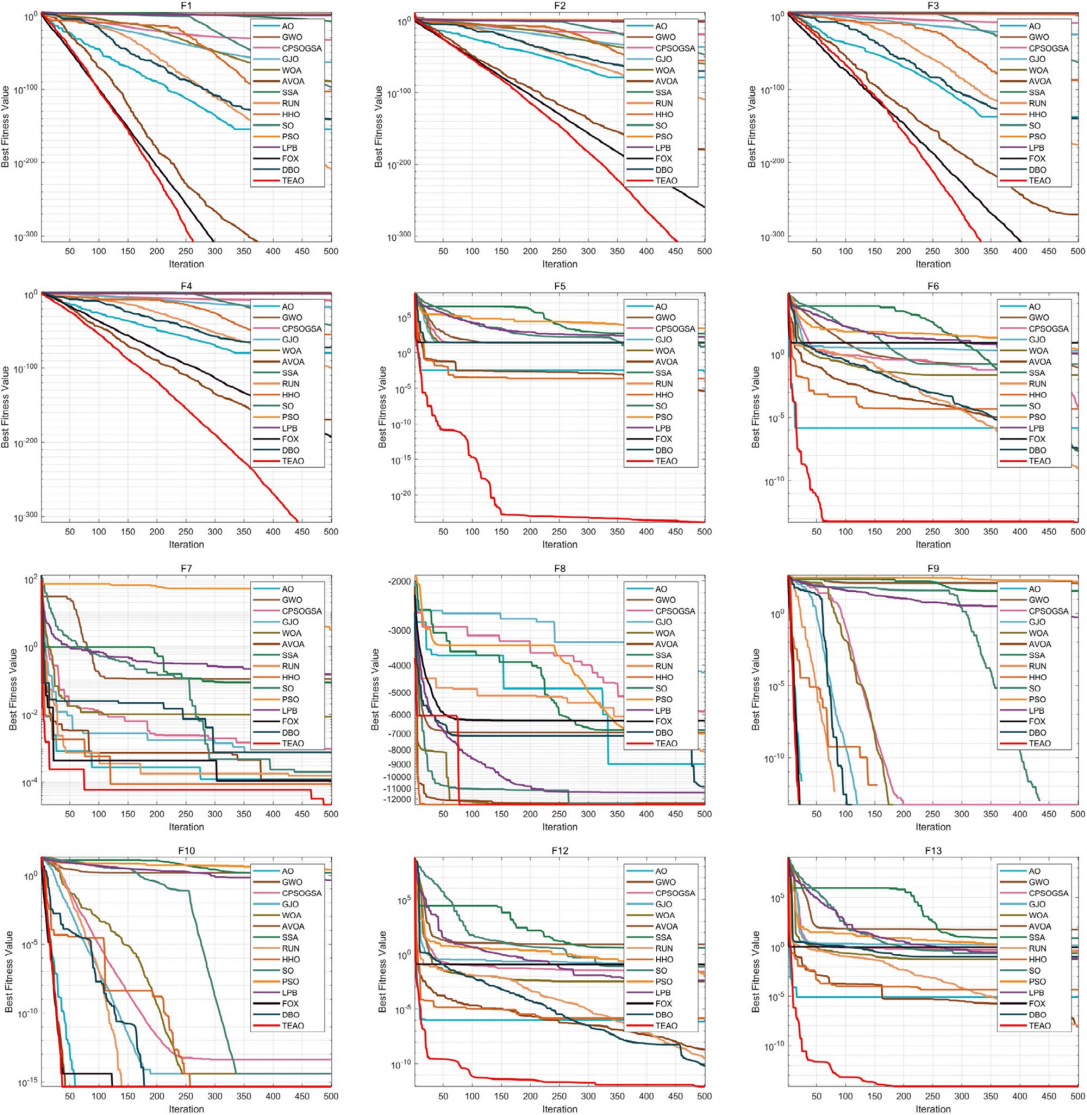
Comparison of convergence curves of 15 algorithms on benchmark function (Dim = 30).

**Figure 9:**
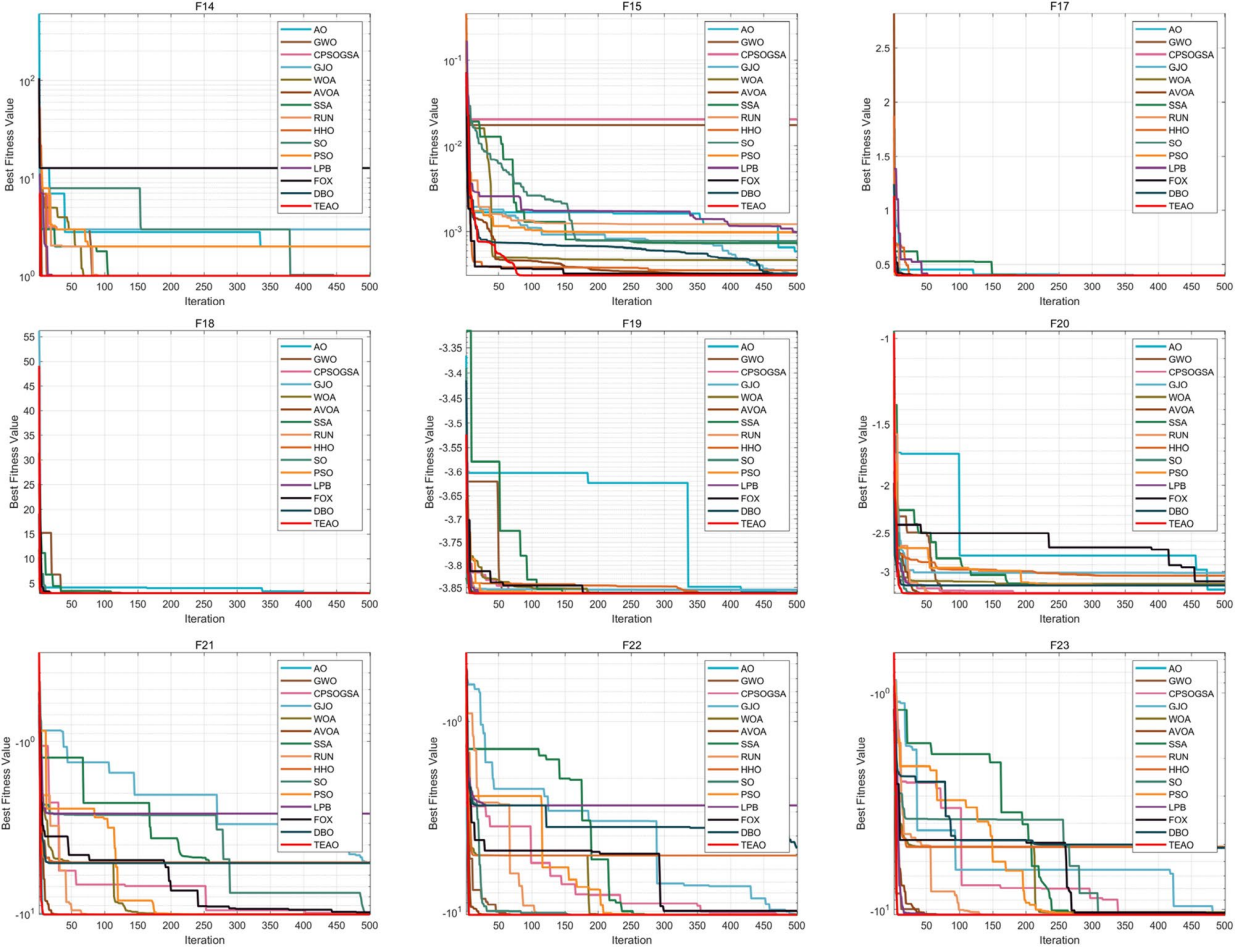
Comparison of convergence curves of 15 algorithms on benchmark function (Dim = Fixed dimension).

When validating the 23 classical benchmark functions, experiments were conducted in dimensions of 30, 100, 500, and 1000 for F1–F13, as well as fixed dimensions for F14–F23. The convergence curves for the selected functions are displayed in Figs. 8 and 9. Results for different dimensions are presented in Tables 6, 7, 8, 9 and 10, 'Ave' represents the mean value, 'Std' indicates the standard deviation, and we highlight the optimal value of the experimental results in bold.

**Table 6:** The experimental results of 15 algorithms on classical test functions (Dim = 30).

ID		AO	GWO	CPSOGSA	GJO	WOA	AVOA	SSA	RUN	HHO	SO	PSO	LPB	FOX	DBO	TEAO
F1	Ave	4.64E-142	2.25E + 01	1.95E-33	2.13E-62	1.30E-81	**0.00E + 00**	2.02E-08	2.30E-200	6.38E-99	7.62E-97	1.69E + 00	1.39E + 00	**0.00E + 00**	2.07E-125	**0.00E + 00**
	Std	8.92E-146	4.54E + 01	1.94E-33	1.43E-60	1.53E-82	**0.00E + 00**	6.24E-09	**0.00E + 00**	5.67E-99	4.98E-96	7.78E-01	8.17E-01	**0.00E + 00**	2.27E-117	**0.00E + 00**
F2	Ave	7.12E-62	5.11E + 01	5.85E-20	2.15E-36	2.94E-53	1.59E-167	8.54E-01	2.74E-107	3.43E-54	1.28E-45	3.74E + 00	2.91E-01	8.74E-290	2.18E-55	**0.00E + 00**
	Std	3.90E-61	4.68E + 01	5.15E-20	3.04E-36	1.49E-52	**0.00E + 00**	6.94E-01	1.45E-106	9.78E-54	2.02E-45	1.01E + 00	7.56E-02	**0.00E + 00**	1.16E-54	**0.00E + 00**
F3	Ave	3.64E-118	5.14E + 03	1.39E-07	1.30E-17	2.78E + 04	2.00E-264	6.11E + 02	1.80E-166	1.72E-84	4.02E-61	1.23E + 02	5.39E + 03	**0.00E + 00**	7.86E-49	**0.00E + 00**
	Std	1.99E-117	2.13E + 03	4.65E-07	7.14E-17	1.08E + 04	**0.00E + 00**	4.06E + 02	**0.00E + 00**	9.41E-84	1.92E-60	3.11E + 01	2.74E + 03	**0.00E + 00**	4.30E-48	**0.00E + 00**
F4	Ave	9.59E-77	3.34E + 01	1.84E-08	1.42E-18	3.63E + 01	1.44E-163	6.63E + 00	1.30E-92	1.19E-50	3.18E-42	1.79E + 00	4.52E + 00	1.02E-288	1.31E-52	**0.00E + 00**
	Std	2.90E-76	6.08E + 00	1.72E-08	5.24E-18	3.48E + 01	**0.00E + 00**	3.39E + 00	6.14E-92	6.15E-50	4.53E-42	1.93E-01	1.02E + 00	**0.00E + 00**	5.08E-52	**0.00E + 00**
F5	Ave	**5.58E-03**	3.46E + 01	2.66E + 01	2.74E + 01	2.75E + 01	4.38E + 00	1.10E + 02	2.33E + 01	5.64E-03	2.25E + 01	7.33E + 02	2.90E + 02	2.88E + 01	2.51E + 01	**1.45E-05**
	Std	1.99E-02	2.81E + 01	6.93E-01	8.04E-01	5.39E-01	**1.22E-05**	1.11E + 02	1.44E + 00	6.07E-03	1.30E + 01	4.16E + 02	2.47E + 02	1.19E-01	1.91E-01	9.96E + 00
F6	Ave	3.68E-05	3.95E + 01	4.61E-01	2.11E + 00	7.22E-02	2.82E-08	2.04E-08	1.16E-09	6.71E-05	1.79E-01	1.93E + 00	1.29E + 00	2.15E + 00	1.45E-08	**3.81E-12**
	Std	6.32E-05	5.39E + 01	2.96E-01	4.80E-01	7.59E-02	1.37E-08	5.69E-09	3.45E-10	9.79E-05	1.68E-01	9.39E-01	5.90E-01	2.46E + 00	1.85E-08	**2.07E-11**
F7	Ave	**6.11E-05**	1.16E-01	1.37E-03	3.82E-04	2.94E-03	7.77E-05	9.10E-02	1.67E-04	1.27E-04	2.47E-04	7.88E + 00	8.44E-02	8.54E-05	2.53E-03	8.53E-05
	Std	**4.48E-05**	3.65E-02	5.11E-04	3.45E-04	3.32E-03	8.29E-05	4.05E-02	1.06E-04	1.33E-04	1.92E-04	5.42E + 00	3.47E-02	6.28E-05	2.00E-03	8.87E-05
F8	Ave	− 8.74E + 03	− 6.38E + 03	− 6.27E + 03	− 4.34E + 03	− 1.07E + 04	− 1.24E + 04	− 7.55E + 03	− 8.10E + 03	− **1.26E + 04**	− 1.25E + 04	− 6.72E + 03	− 1.09E + 04	− 7.28E + 03	− 9.27E + 03	− 9.38E + 03
	Std	3.54E + 03	5.64E + 02	7.71E + 02	9.29E + 02	1.82E + 03	3.67E + 02	7.21E + 02	5.34E + 02	**5.43E + 01**	1.34E + 02	9.24E + 02	3.08E + 02	6.26E + 02	1.60E + 03	2.70E + 03
F9	Ave	**0.00E + 00**	1.31E + 02	1.55E + 00	**0.00E + 00**	5.68E-15	**0.00E + 00**	4.86E + 01	**0.00E + 00**	**0.00E + 00**	4.87E + 00	1.47E + 02	1.54E + 00	**0.00E + 00**	6.82E + 00	**0.00E + 00**
	Std	**0.00E + 00**	2.93E + 01	2.77E + 00	**0.00E + 00**	2.29E-14	**0.00E + 00**	1.57E + 01	**0.00E + 00**	**0.00E + 00**	9.84E + 00	2.81E + 01	9.16E-01	**0.00E + 00**	2.16E + 01	**0.00E + 00**
F10	Ave	**4.44E-16**	3.35E + 00	4.30E-14	6.37E-15	4.47E-15	**4.44E-16**	2.12E + 00	**4.44E-16**	**4.44E-16**	4.00E-15	2.29E + 00	3.58E-01	**4.44E-16**	5.63E-16	**4.44E-16**
	Std	**0.00E + 00**	3.51E + 00	4.12E-15	1.70E-15	2.76E-15	**0.00E + 00**	6.76E-01	**0.00E + 00**	**0.00E + 00**	**0.00E + 00**	4.31E-01	1.20E-01	**0.00E + 00**	6.49E-16	**0.00E + 00**
F11	Ave	**0.00E + 00**	5.10E + 00	3.37E-03	**0.00E + 00**	1.60E-02	**0.00E + 00**	9.70E-03	**0.00E + 00**	**0.00E + 00**	1.36E-02	9.56E-02	8.94E-01	**0.00E + 00**	4.58E-03	**0.00E + 00**
	Std	**0.00E + 00**	2.32E + 00	7.57E-03	**0.00E + 00**	5.41E-02	**0.00E + 00**	1.42E-02	**0.00E + 00**	**0.00E + 00**	3.10E-02	4.15E-02	1.50E-01	**0.00E + 00**	2.28E-02	**0.00E + 00**
F12	Ave	7.72E-07	9.23E + 00	2.43E-02	1.84E-01	6.26E-03	1.83E-09	5.20E + 00	5.48E-10	2.03E-06	1.21E-01	3.91E-02	9.84E-03	1.11E-01	9.71E-09	**4.88E-11**
	Std	1.27E-06	2.85E + 00	1.47E-02	1.06E-01	5.75E-03	1.12E-09	1.57E + 00	**1.83E-10**	2.15E-06	2.18E-01	4.44E-02	1.88E-02	5.39E-02	4.19E-08	1.95E-10
F13	Ave	1.78E-05	3.59E + 01	4.25E-01	1.43E + 00	2.14E-01	1.06E-08	4.65E + 00	3.63E-03	2.58E-05	3.79E-01	3.93E-01	8.81E-02	1.01E + 00	1.11E-01	**1.08E-09**
	Std	2.83E-05	1.68E + 01	2.18E-01	2.45E-01	1.36E-01	1.37E-08	7.56E + 00	5.91E-03	2.42E-05	4.93E-01	1.95E-01	4.03E-02	8.19E-01	1.44E-01	**5.66E-09**

**Table 7:** The experimental results of 15 algorithms on classical test functions (Dim = Fixed dimension).

ID		AO	GWO	CPSOGSA	GJO	WOA	AVOA	SSA	RUN	HHO	SO	PSO	LPB	FOX	DBO	TEAO
F14	Ave	1.82E + 00	2.74E + 00	3.15E + 00	4.33E + 00	1.85E + 00	1.06E + 00	1.13E + 00	2.44E + 00	1.36E + 00	1.06E + 00	1.89E + 00	9.98E-01	8.17E + 00	**9.98E-01**	2.63E + 00
	Std	1.87E + 00	2.39E + 00	3.57E + 00	4.04E + 00	1.87E + 00	3.62E-01	4.31E-01	2.46E + 00	9.87E-01	3.62E-01	1.25E + 00	1.43E-10	5.61E + 00	**1.30E-16**	3.50E + 00
F15	Ave	4.51E-04	9.31E-03	3.73E-03	3.93E-04	7.31E-04	3.37E-04	1.45E-03	6.74E-04	3.62E-04	4.45E-04	8.37E-04	3.60E-03	3.68E-04	8.27E-04	**3.07E-04**
	Std	9.10E-05	2.31E-02	7.57E-03	1.71E-04	4.98E-04	6.47E-05	3.58E-03	4.56E-04	1.83E-04	1.91E-04	1.59E-04	6.32E-03	1.63E-04	3.72E-04	**1.07E-13**
F16	Ave	− 1.03E + 00	− 1.03E + 00	− 1.03E + 00	− 1.03E + 00	− 1.03E + 00	− **1.03E + 00**	− 1.03E + 00	− 1.03E + 00	− 1.03E + 00	− **1.03E + 00**	− **1.03E + 00**	− 1.03E + 00	− 1.03E + 00	− **1.03E + 00**	− **1.03E + 00**
	Std	3.23E-04	5.45E-16	1.14E-08	1.28E-07	5.11E-10	**4.48E-16**	1.58E-14	3.79E-13	5.40E-11	5.90E-16	5.13E-16	2.88E-06	2.18E-06	6.39E-16	5.68E-16
F17	Ave	3.98E-01	**3.98E-01**	3.98E-01	3.98E-01	3.98E-01	**3.98E-01**	3.98E-01	3.98E-01	3.98E-01	**3.98E-01**	**3.98E-01**	3.98E-01	3.98E-01	**3.98E-01**	**3.98E-01**
	Std	1.86E-04	**0.00E + 00**	1.94E-06	1.12E-05	2.77E-06	**0.00E + 00**	4.41E-14	1.56E-11	2.19E-06	**0.00E + 00**	**0.00E + 00**	1.56E-09	1.63E-07	**0.00E + 00**	**0.00E + 00**
F18	Ave	3.01E + 00	**3.00E + 00**	3.00E + 00	3.00E + 00	3.00E + 00	3.00E + 00	3.00E + 00	3.00E + 00	3.00E + 00	3.00E + 00	3.00E + 00	3.00E + 00	3.00E + 00	**3.00E + 00**	**3.00E + 00**
	Std	1.67E-02	2.12E-15	1.43E-05	9.67E-07	7.77E-06	1.00E-06	2.98E-13	3.67E-13	1.10E-07	2.17E-15	4.48E-15	1.89E-05	1.28E-04	**1.84E-15**	5.56E-15
F19	Ave	− 3.86E + 00	− 3.86E + 00	− 3.86E + 00	− 3.86E + 00	− 3.86E + 00	− 3.86E + 00	− 3.86E + 00	− 3.86E + 00	− 3.86E + 00	− **3.86E + 00**	− **3.86E + 00**	− 3.86E + 00	− 3.86E + 00	− 3.86E + 00	− **3.86E + 00**
	Std	4.86E-03	2.40E-15	1.64E-03	3.88E-03	2.29E-03	5.98E-14	9.92E-14	2.10E-09	1.49E-03	2.57E-15	**2.13E-15**	8.73E-07	1.13E-03	2.28E-03	2.47E-15
F20	Ave	− 3.19E + 00	− 3.28E + 00	− 3.24E + 00	− 3.18E + 00	− 3.21E + 00	− 3.28E + 00	− 3.22E + 00	− 3.27E + 00	− 3.15E + 00	− **3.32E + 00**	− 3.27E + 00	− 3.27E + 00	− 2.97E + 00	− 3.23E + 00	− 3.25E + 00
	Std	8.39E-02	5.83E-02	8.20E-02	1.17E-01	1.12E-01	5.87E-02	5.17E-02	5.99E-02	1.08E-01	**9.34E-15**	5.92E-02	5.99E-02	8.48E-02	7.78E-02	5.83E-02
F21	Ave	− 1.01E + 01	− 8.72E + 00	− 9.31E + 00	− 8.43E + 00	− 9.22E + 00	− **1.02E + 01**	− 8.31E + 00	− 1.02E + 01	− 5.05E + 00	− 1.01E + 01	− 7.38E + 00	− 5.56E + 00	− 8.50E + 00	− 7.08E + 00	− 1.02E + 01
	Std	5.40E-03	2.70E + 00	1.92E + 00	2.72E + 00	2.15E + 00	**7.08E-15**	2.94E + 00	1.04E-09	2.91E-03	1.19E-01	2.91E + 00	3.41E + 00	2.17E + 00	2.55E + 00	3.42E-09
F22	Ave	− 1.04E + 01	− 8.57E + 00	− 1.04E + 01	− 9.57E + 00	− 8.12E + 00	− **1.04E + 01**	− 9.46E + 00	− 1.02E + 01	− 5.43E + 00	− 1.03E + 01	− 9.65E + 00	− 6.93E + 00	− 9.00E + 00	− 7.12E + 00	− 1.04E + 01
	Std	6.49E− 03	3.11E + 00	6.21E-04	2.17E + 00	2.89E + 00	**3.76E-15**	2.47E + 00	9.70E-01	1.33E + 00	3.47E-01	1.96E + 00	3.62E + 00	2.23E + 00	2.75E + 00	4.57E-09
F23	Ave	− 1.05E + 01	− 8.71E + 00	− 1.05E + 01	− 1.02E + 01	− 8.00E + 00	− **1.05E + 01**	− 9.30E + 00	− 1.05E + 01	− 5.48E + 00	− 1.04E + 01	− 1.05E + 01	− 6.70E + 00	− 9.35E + 00	− 9.24E + 00	− 1.05E + 01
	Std	9.14E-03	3.10E + 00	7.61E-04	1.36E + 00	3.21E + 00	**2.97E-15**	2.83E + 00	3.96E-10	1.35E + 00	4.96E-01	2.04E-09	3.73E + 00	1.98E + 00	2.40E + 00	4.01E-09

**Table 8:** The experimental results of 15 algorithms on classical test functions (Dim = 100).

ID		AO	GWO	CPSOGSA	GJO	WOA	AVOA	SSA	RUN	HHO	SO	PSO	LPB	FOX	DBO	TEAO
F1	Ave	6.99E-144	1.45E + 04	4.29E-15	7.47E-31	3.11E-83	**0.00E + 00**	3.27E + 02	6.58E-180	9.92E-98	3.54E-84	1.65E + 02	2.14E + 02	**0.00E + 00**	5.52E-116	**0.00E + 00**
	Std	3.83E-143	3.81E + 03	2.46E-15	1.38E-30	1.12E-82	**0.00E + 00**	1.20E + 02	**0.00E + 00**	5.04E-97	1.11E-83	2.95E + 01	4.43E + 01	**0.00E + 00**	2.65E-115	**0.00E + 00**
F2	Ave	3.28E-72	3.33E + 02	1.42E-09	2.42E-19	1.14E-52	1.95E-165	3.05E + 01	9.15E-101	2.14E-51	1.66E-36	1.91E + 02	6.40E + 01	1.72E-232	3.86E-55	**0.00E + 00**
	Std	1.80E-71	2.64E + 01	4.27E-10	1.60E-19	3.01E-52	**0.00E + 00**	4.95E + 00	4.70E-100	7.35E-51	1.80E-36	4.55E + 01	1.18E + 01	**0.00E + 00**	2.11E-54	**0.00E + 00**
F3	Ave	4.66E-142	1.34E + 05	1.35E + 02	9.58E-03	9.13E + 05	4.54E-236	2.45E + 04	1.24E-146	2.13E-73	6.48E-35	1.47E + 04	8.76E + 04	**0.00E + 00**	1.12E-15	**0.00E + 00**
	Std	2.55E-141	2.30E + 04	1.45E + 02	4.21E-02	1.93E + 05	**0.00E + 00**	1.32E + 04	6.82E-146	1.17E-72	3.55E-34	2.73E + 03	1.03E + 04	**0.00E + 00**	6.15E-15	**0.00E + 00**
F4	Ave	1.16E-60	7.76E + 01	1.82E-01	2.03E + 00	8.09E + 01	1.71E-166	2.18E + 01	3.24E-85	3.31E-51	1.20E-37	1.11E + 01	5.86E + 01	6.00E-231	6.10E-52	**0.00E + 00**
	Std	6.36E-60	1.52E + 01	1.76E-01	3.32E + 00	1.44E + 01	**0.00E + 00**	2.83E + 00	1.64E-84	1.03E-50	1.85E-37	1.17E + 00	5.57E + 00	**0.00E + 00**	2.87E-51	**0.00E + 00**
F5	Ave	2.89E + 01	4.26E + 05	9.75E + 01	9.81E + 01	9.79E + 01	**1.21E-04**	2.43E + 04	9.50E + 01	9.98E-03	6.10E + 01	2.13E + 05	2.12E + 04	9.86E + 01	9.61E + 01	**6.23E-03**
	Std	4.49E + 01	2.81E + 05	8.60E-01	5.70E-01	3.43E-01	**1.35E-04**	1.18E + 04	1.96E + 00	1.55E-02	4.14E + 01	4.49E + 04	1.94E + 04	3.87E-01	4.05E-01	1.15E-02
F6	Ave	1.55E-04	1.28E + 04	8.37E + 00	1.59E + 01	1.77E + 00	4.63E-05	3.56E + 02	2.23E-05	2.34E-04	1.38E + 01	1.64E + 02	2.29E + 02	1.00E + 01	2.08E + 00	**5.85E-11**
	Std	2.83E-04	4.01E + 03	6.74E-01	8.84E-01	4.45E-01	2.49E-05	1.45E + 02	6.67E-05	3.43E-04	1.02E + 01	2.44E + 01	5.14E + 01	7.67E + 00	5.27E-01	**3.20E-10**
F7	Ave	9.81E-05	4.38E + 00	4.66E-03	9.51E-04	3.29E-03	7.37E-05	1.44E + 00	1.56E-04	9.46E-05	1.35E-04	1.49E + 03	6.03E-01	8.59E-05	1.79E-03	**5.27E-05**
	Std	1.29E-04	1.48E + 00	2.03E-03	5.29E-04	3.41E-03	**5.40E-05**	2.82E-01	1.13E-04	9.33E-05	1.21E-04	2.24E + 02	1.26E-01	8.48E-05	1.21E-03	6.12E-05
F8	Ave	− 1.31E + 04	− 1.64E + 04	− 1.64E + 04	− 1.08E + 04	− 3.70E + 04	− 4.14E + 04	− 2.31E + 04	− 2.34E + 04	− **4.19E + 04**	− 4.17E + 04	− 2.16E + 04	− 2.77E + 04	− 2.27E + 04	− 2.75E + 04	− 2.78E + 04
	Std	6.03E + 03	1.40E + 03	3.79E + 03	4.48E + 03	5.08E + 03	1.22E + 03	1.78E + 03	2.26E + 03	**2.33E + 02**	7.32E + 02	2.44E + 03	1.26E + 03	2.27E + 03	5.30E + 03	1.06E + 04
F9	Ave	**0.00E + 00**	4.67E + 02	6.63E + 00	7.58E-15	**0.00E + 00**	**0.00E + 00**	1.69E + 02	**0.00E + 00**	**0.00E + 00**	6.98E + 00	1.12E + 03	4.02E + 02	**0.00E + 00**	6.63E + 00	**0.00E + 00**
	Std	**0.00E + 00**	4.03E + 01	5.74E + 00	4.15E-14	**0.00E + 00**	**0.00E + 00**	4.02E + 01	**0.00E + 00**	**0.00E + 00**	2.38E + 01	1.20E + 02	5.47E + 01	**0.00E + 00**	3.63E + 01	**0.00E + 00**
F10	Ave	**4.44E-16**	1.69E + 01	6.98E-09	3.23E-14	3.52E-15	**4.44E-16**	7.58E + 00	**4.44E-16**	**4.44E-16**	4.00E-15	6.57E + 00	5.62E + 00	2.10E-15	**4.44E-16**	**4.44E-16**
	Std	**0.00E + 00**	1.83E + 00	2.57E-09	4.71E-15	2.76E-15	**0.00E + 00**	1.14E + 00	**0.00E + 00**	**0.00E + 00**	**0.00E + 00**	3.39E-01	8.64E-01	1.80E-15	**0.00E + 00**	**0.00E + 00**
F11	Ave	**0.00E + 00**	3.38E + 02	2.83E-03	**0.00E + 00**	**0.00E + 00**	**0.00E + 00**	3.98E + 00	**0.00E + 00**	**0.00E + 00**	**0.00E + 00**	1.00E + 00	3.01E + 00	**0.00E + 00**	**0.00E + 00**	**0.00E + 00**
	Std	**0.00E + 00**	9.64E + 01	6.51E-03	**0.00E + 00**	**0.00E + 00**	**0.00E + 00**	9.19E-01	**0.00E + 00**	**0.00E + 00**	**0.00E + 00**	3.84E-02	5.09E-01	**0.00E + 00**	**0.00E + 00**	**0.00E + 00**
F12	Ave	1.34E-06	1.49E + 05	2.43E-01	5.46E-01	2.05E-02	2.68E-07	1.64E + 01	1.93E-08	7.91E-07	9.62E-02	9.58E + 00	8.87E + 01	2.36E-01	1.99E-02	**7.18E-11**
	Std	4.46E-06	1.42E + 05	7.58E-02	8.19E-02	1.26E-02	7.24E-07	4.95E + 00	4.88E-09	8.87E-07	2.77E-01	3.50E + 00	1.01E + 02	3.70E-01	6.71E-03	**3.72E-10**
F13	Ave	1.53E-05	3.78E + 06	6.05E + 00	8.10E + 00	1.64E + 00	4.59E-08	1.92E + 02	1.18E-02	4.61E-05	2.72E + 00	1.84E + 02	8.39E + 02	4.07E + 00	7.12E + 00	**1.84E-11**
	Std	2.56E-05	2.02E + 06	3.43E-01	2.76E-01	5.85E-01	4.84E-08	2.85E + 01	9.94E-03	6.18E-05	3.93E + 00	9.78E + 01	9.08E + 02	1.66E + 00	8.89E-01	**7.11E-11**

**Table 9:** The experimental results of 15 algorithms on classical test functions (Dim = 500).

ID		AO	GWO	CPSOGSA	GJO	WOA	AVOA	SSA	RUN	HHO	SO	PSO	LPB	FOX	DBO	TEAO
F1	Ave	3.21E-118	4.44E + 05	1.71E-04	7.95E-14	1.46E-80	**0.00E + 00**	5.75E + 04	2.42E-168	1.13E-99	8.49E-72	1.02E + 04	1.18E + 05	**0.00E + 00**	1.24E-104	**0.00E + 00**
	Std	1.76E-117	3.55E + 04	6.75E-05	4.72E-14	7.41E-80	**0.00E + 00**	3.95E + 03	**0.00E + 00**	4.38E-99	2.75E-71	4.52E + 02	9.01E + 03	**0.00E + 00**	6.81E-104	**0.00E + 00**
F2	Ave	7.18E-68	8.11E + 260	3.00E-03	1.82E-09	8.66E-52	3.11E-165	4.22E + 02	1.18E-96	8.10E-52	1.33E-31	6.00E + 144	2.23E + 100	1.25E-195	1.94E-62	**0.00E + 00**
	Std	3.93E-67	Inf	4.95E-04	7.31E-10	3.95E-51	**0.00E + 00**	1.66E + 01	6.00E-96	3.98E-51	1.20E-31	3.28E + 145	1.22E + 101	**0.00E + 00**	7.61E-62	**0.00E + 00**
F3	Ave	2.19E-107	3.75E + 06	2.41E + 05	1.50E + 04	2.48E + 07	4.12E-213	7.64E + 05	2.17E-144	4.77E-48	4.35E-09	5.72E + 05	2.45E + 06	**0.00E + 00**	7.11E-04	**0.00E + 00**
	Std	1.20E-106	7.36E + 05	8.06E + 04	1.81E + 04	7.10E + 06	**0.00E + 00**	3.36E + 05	1.19E-143	2.49E-47	2.38E-08	1.26E + 05	2.84E + 05	**0.00E + 00**	3.89E-03	**0.00E + 00**
F4	Ave	1.01E-74	9.84E + 01	6.02E + 01	8.11E + 01	7.73E + 01	8.91E-164	3.24E + 01	8.44E-81	1.25E-51	1.01E-34	2.67E + 01	9.25E + 01	2.31E-196	4.45E-35	**0.00E + 00**
	Std	4.45E-74	1.29E + 00	5.43E + 00	4.04E + 00	2.35E + 01	**0.00E + 00**	2.25E + 00	3.36E-80	4.41E-51	1.21E-34	1.20E + 00	1.32E + 00	**0.00E + 00**	2.18E-34	**0.00E + 00**
F5	Ave	**2.44E-02**	3.28E + 08	4.97E + 02	4.98E + 02	4.95E + 02	1.98E + 02	1.42E + 07	4.93E + 02	7.13E-02	3.65E + 02	8.09E + 07	1.79E + 08	4.97E + 02	4.98E + 02	**4.02E-03**
	Std	3.18E-02	5.00E + 07	1.76E-01	1.90E-01	3.33E-01	2.46E + 02	2.18E + 06	1.64E + 00	9.72E-02	1.98E + 02	7.44E + 06	3.05E + 07	1.80E + 00	1.97E-01	**4.96E-03**
F6	Ave	3.39E-04	4.38E + 05	8.68E + 01	1.07E + 02	1.60E + 01	3.84E-02	5.80E + 04	1.03E + 00	8.33E-04	5.57E + 01	1.04E + 04	1.21E + 05	4.35E + 01	8.65E + 01	**3.85E-11**
	Std	7.32E-04	3.18E + 04	1.90E + 00	1.67E + 00	4.15E + 00	1.12E-01	4.07E + 03	1.52E-01	1.26E-03	5.42E + 01	4.54E + 02	9.46E + 03	2.81E + 01	4.49E + 00	**2.03E-10**
F7	Ave	**5.77E-05**	2.38E + 03	3.01E-02	4.38E-03	2.07E-03	8.37E-05	1.13E + 02	2.53E-04	9.59E-05	1.63E-04	5.62E + 04	1.27E + 03	8.54E-05	1.43E-03	1.04E-04
	Std	**5.65E-05**	4.33E + 02	6.85E-03	2.44E-03	1.67E-03	1.06E-04	1.55E + 01	1.88E-04	8.31E-05	1.24E-04	2.19E + 03	2.54E + 02	9.43E-05	1.18E-03	9.77E-05
F8	Ave	− 4.21E + 04	− 4.02E + 04	− 5.77E + 04	− 2.87E + 04	− 1.88E + 05	− 2.07E + 05	− 6.81E + 04	− 9.27E + 04	− **2.09E + 05**	− 2.08E + 05	− 8.83E + 04	− 8.54E + 04	− 9.23E + 04	− 1.50E + 05	− 1.20E + 05
	Std	1.49E + 04	3.04E + 03	1.22E + 04	1.51E + 04	2.69E + 04	3.46E + 03	4.16E + 03	1.39E + 04	**1.08E + 01**	2.59E + 03	1.62E + 04	3.93E + 03	1.67E + 04	3.54E + 04	7.79E + 04
F9	Ave	**0.00E + 00**	3.53E + 03	5.76E + 01	4.67E-12	3.03E-14	**0.00E + 00**	2.77E + 03	**0.00E + 00**	**0.00E + 00**	1.71E + 01	7.93E + 03	5.22E + 03	**0.00E + 00**	**0.00E + 00**	**0.00E + 00**
	Std	**0.00E + 00**	2.42E + 02	1.91E + 01	1.14E-12	1.66E-13	**0.00E + 00**	1.18E + 02	**0.00E + 00**	**0.00E + 00**	9.15E + 01	2.19E + 02	1.70E + 02	**0.00E + 00**	**0.00E + 00**	**0.00E + 00**
F10	Ave	**4.44E-16**	1.99E + 01	5.95E-04	9.57E-09	3.40E-15	**4.44E-16**	1.26E + 01	**4.44E-16**	**4.44E-16**	4.12E-15	1.37E + 01	2.01E + 01	3.52E-15	**4.44E-16**	**4.44E-16**
	Std	**0.00E + 00**	1.57E-01	1.06E-04	3.52E-09	2.30E-15	**0.00E + 00**	2.46E-01	**0.00E + 00**	**0.00E + 00**	6.49E-16	1.98E-01	1.61E-01	1.23E-15	**0.00E + 00**	**0.00E + 00**
F11	Ave	**0.00E + 00**	5.76E + 03	2.04E-03	1.46E-14	**0.00E + 00**	**0.00E + 00**	5.26E + 02	**0.00E + 00**	**0.00E + 00**	**0.00E + 00**	8.17E + 00	1.06E + 03	**0.00E + 00**	**0.00E + 00**	**0.00E + 00**
	Std	**0.00E + 00**	4.70E + 02	1.11E-02	1.11E-14	**0.00E + 00**	**0.00E + 00**	3.20E + 01	**0.00E + 00**	**0.00E + 00**	**0.00E + 00**	6.29E-01	7.53E + 01	**0.00E + 00**	**0.00E + 00**	**0.00E + 00**
F12	Ave	1.66E-07	5.09E + 08	6.85E-01	9.01E-01	3.63E-02	1.33E-05	4.16E + 04	9.46E-04	6.97E-07	1.57E-01	1.58E + 06	1.93E + 08	3.65E-01	5.47E-01	**4.42E-14**
	Std	1.97E-07	1.08E + 08	4.18E-02	2.85E-02	1.53E-02	7.06E-05	4.78E + 04	1.40E-04	7.74E-07	3.79E-01	4.17E + 05	3.66E + 07	4.30E-01	4.06E-02	**1.42E-13**
F13	Ave	8.02E-05	1.47E + 09	4.83E + 01	4.75E + 01	1.05E + 01	3.42E-07	7.01E + 06	2.25E + 00	1.69E-04	1.07E + 01	1.57E + 07	5.97E + 08	2.33E + 01	4.90E + 01	**1.84E-10**
	Std	1.34E-04	1.93E + 08	1.22E + 00	3.53E-01	3.41E + 00	3.16E-07	2.35E + 06	6.36E-01	3.43E-04	1.80E + 01	3.24E + 06	1.43E + 08	9.55E + 00	3.38E-01	**7.81E-10**

**Table 10:** The experimental results of 15 algorithms on classical test functions (Dim = 1000).

ID		AO	GWO	CPSOGSA	GJO	WOA	AVOA	SSA	RUN	HHO	SO	PSO	LPB	FOX	DBO	TEAO
F1	Ave	8.12E-128	1.32E + 06	5.07E-02	5.53E-10	7.04E-80	**0.00E + 00**	1.54E + 05	1.18E-167	2.43E-101	2.36E-68	5.67E + 04	7.47E + 05	**0.00E + 00**	1.30E-107	**0.00E + 00**
	Std	4.45E-127	7.31E + 04	1.44E-02	3.70E-10	3.26E-79	**0.00E + 00**	7.30E + 03	**0.00E + 00**	8.60E-101	5.37E-68	1.66E + 03	4.07E + 04	**0.00E + 00**	7.13E-107	**0.00E + 00**
F2	Ave	1.15E-76	Inf	2.78E-01	2.15E-07	6.68E-52	**0.00E + 00**	9.65E + 02	1.93E-93	6.76E-52	7.56E-28	2.86E + 27	2.57E + 03	**0.00E + 00**	7.26E-59	**0.00E + 00**
	Std	4.57E-76	NaN	1.71E-01	6.90E-08	2.14E-51	**0.00E + 00**	2.14E + 01	7.74E-93	2.23E-51	2.22E-27	1.57E + 28	7.09E + 01	**0.00E + 00**	3.94E-58	**0.00E + 00**
F3	Ave	4.52E-104	1.46E + 07	1.16E + 06	1.91E + 05	1.11E + 08	3.36E-207	3.22E + 06	2.30E-141	9.31E-25	1.97E-08	2.20E + 06	9.39E + 06	**0.00E + 00**	3.69E-02	**0.00E + 00**
	Std	2.48E-103	2.28E + 06	1.81E + 05	1.41E + 05	2.83E + 07	**0.00E + 00**	1.34E + 06	1.14E-140	3.89E-24	1.08E-07	4.40E + 05	9.91E + 05	**0.00E + 00**	2.02E-01	**0.00E + 00**
F4	Ave	1.42E-70	9.93E + 01	7.67E + 01	8.85E + 01	7.99E + 01	3.77E-165	3.68E + 01	1.75E-80	1.72E-50	8.31E-34	3.18E + 01	9.69E + 01	3.48E-188	1.37E-20	**0.00E + 00**
	Std	7.80E-70	7.00E-01	3.82E + 00	2.55E + 00	2.20E + 01	**0.00E + 00**	2.74E + 00	5.97E-80	6.59E-50	1.22E-33	1.14E + 00	6.26E-01	**0.00E + 00**	7.52E-20	**0.00E + 00**
F5	Ave	**1.35E-01**	1.60E + 09	1.01E + 03	9.98E + 02	9.92E + 02	9.89E + 01	4.92E + 07	9.90E + 02	1.66E-01	7.53E + 02	5.79E + 08	1.97E + 09	9.95E + 02	9.98E + 02	**1.09E-02**
	Std	2.46E-01	1.75E + 08	3.80E + 00	9.09E-02	6.71E-01	3.02E + 02	4.21E + 06	1.36E + 00	2.01E-01	3.92E + 02	4.95E + 07	2.02E + 08	3.66E + 00	2.62E-01	**1.12E-02**
F6	Ave	2.01E-04	1.32E + 06	1.94E + 02	2.28E + 02	3.85E + 01	1.70E-01	1.55E + 05	4.79E + 00	2.42E-03	1.65E + 02	5.63E + 04	7.59E + 05	1.34E + 02	2.05E + 02	**1.80E-23**
	Std	2.72E-04	5.98E + 04	2.66E + 00	1.57E + 00	7.94E + 00	5.24E-01	5.79E + 03	3.84E-01	4.91E-03	1.02E + 02	2.13E + 03	4.51E + 04	8.96E + 01	4.34E + 00	**9.86E-23**
F7	Ave	**4.41E-05**	2.31E + 04	8.35E-02	8.81E-03	2.21E-03	1.18E-04	7.08E + 02	2.33E-04	8.91E-05	1.28E-04	2.37E + 05	2.69E + 04	8.11E-05	1.61E-03	8.59E-05
	Std	**2.93E-05**	2.71E + 03	1.79E-02	4.48E-03	2.32E-03	1.25E-04	6.28E + 01	1.83E-04	8.97E-05	9.98E-05	7.65E + 03	2.28E + 03	7.98E-05	1.14E-03	7.05E-05
F8	Ave	− 5.95E + 04	− 6.05E + 04	− 9.65E + 04	− 4.26E + 04	− 3.81E + 05	− 4.13E + 05	− 9.85E + 04	− 1.71E + 05	− **4.19E + 05**	− 4.16E + 05	− 1.36E + 05	− 1.27E + 05	− 1.39E + 05	− 3.22E + 05	− 1.68E + 05
	Std	1.64E + 04	5.96E + 03	1.53E + 04	2.45E + 04	4.90E + 04	7.75E + 03	5.50E + 03	2.94E + 04	**7.42E + 00**	4.94E + 03	1.68E + 04	5.06E + 03	4.33E + 04	4.41E + 04	1.57E + 05
F9	Ave	**0.00E + 00**	8.94E + 03	1.54E + 02	1.49E-10	2.43E-13	**0.00E + 00**	7.01E + 03	**0.00E + 00**	**0.00E + 00**	4.64E + 01	1.67E + 04	1.26E + 04	**0.00E + 00**	**0.00E + 00**	**0.00E + 00**
	Std	**0.00E + 00**	5.46E + 02	2.98E + 01	2.23E-10	9.23E-13	**0.00E + 00**	1.94E + 02	**0.00E + 00**	**0.00E + 00**	2.06E + 02	2.82E + 02	2.72E + 02	**0.00E + 00**	**0.00E + 00**	**0.00E + 00**
F10	Ave	**4.44E-16**	2.01E + 01	7.96E-03	5.92E-07	4.00E-15	**4.44E-16**	1.30E + 01	**4.44E-16**	**4.44E-16**	4.83E-15	1.67E + 01	2.06E + 01	3.88E-15	5.63E-16	**4.44E-16**
	Std	**0.00E + 00**	1.49E-01	1.01E-03	1.92E-07	2.64E-15	**0.00E + 00**	1.74E-01	**0.00E + 00**	**0.00E + 00**	1.53E-15	1.68E-01	6.14E-02	6.49E-16	6.49E-16	**0.00E + 00**
F11	Ave	**0.00E + 00**	1.43E + 04	3.04E-02	5.22E-11	3.70E-18	**0.00E + 00**	1.37E + 03	**0.00E + 00**	**0.00E + 00**	**0.00E + 00**	6.93E + 01	6.88E + 03	**0.00E + 00**	**0.00E + 00**	**0.00E + 00**
	Std	**0.00E + 00**	5.19E + 02	7.14E-02	8.51E-11	2.03E-17	**0.00E + 00**	5.62E + 01	**0.00E + 00**	**0.00E + 00**	**0.00E + 00**	3.61E + 00	3.24E + 02	**0.00E + 00**	**0.00E + 00**	**0.00E + 00**
F12	Ave	4.32E-07	2.60E + 09	9.34E-01	9.96E-01	3.82E-02	1.12E-06	7.57E + 05	2.49E-03	1.24E-06	6.36E-02	2.98E + 07	3.50E + 09	4.01E-01	7.89E-01	**4.24E-14**
	Std	6.75E-07	2.88E + 08	1.34E-01	3.75E-02	1.74E-02	2.51E-06	3.90E + 05	1.51E-04	3.62E-06	2.02E-01	5.18E + 06	4.77E + 08	4.46E-01	3.92E-02	**2.32E-13**
F13	Ave	1.51E-04	7.04E + 09	1.10E + 02	9.79E + 01	1.88E + 01	7.62E-07	3.79E + 07	5.19E + 00	2.12E-04	1.61E + 01	2.11E + 08	8.00E + 09	4.96E + 01	9.93E + 01	**3.27E-14**
	Std	3.74E-04	7.75E + 08	3.65E + 00	4.86E-01	6.13E + 00	1.59E-02	6.72E + 06	8.71E-01	5.36E-04	3.07E + 01	2.26E + 07	8.87E + 08	2.10E + 01	1.94E-01	**3.68E-13**

From Figs. 8 and 9, it is evident that algorithms such as AO, AVOA, DBO, FOX, PSO, and GJO tend to get stuck in local optima during the later iterations, lacking the ability to escape from local optima. In contrast, TEAO maintains a strong exploration capability even in the later stages of iterations. While functions F5, F7, and F15 temporarily converge to local optima during a certain period of iterations, TEAO manages to escape from these local optima in the later iterations and continues to explore deeply, ultimately achieving higher convergence accuracy. This indicates the effectiveness of the new update rules and Levy flight strategy that we introduced. These strategies not only help the algorithm escape from local optima but also enhance the algorithm's convergence speed and precision.

**Figure 10:**
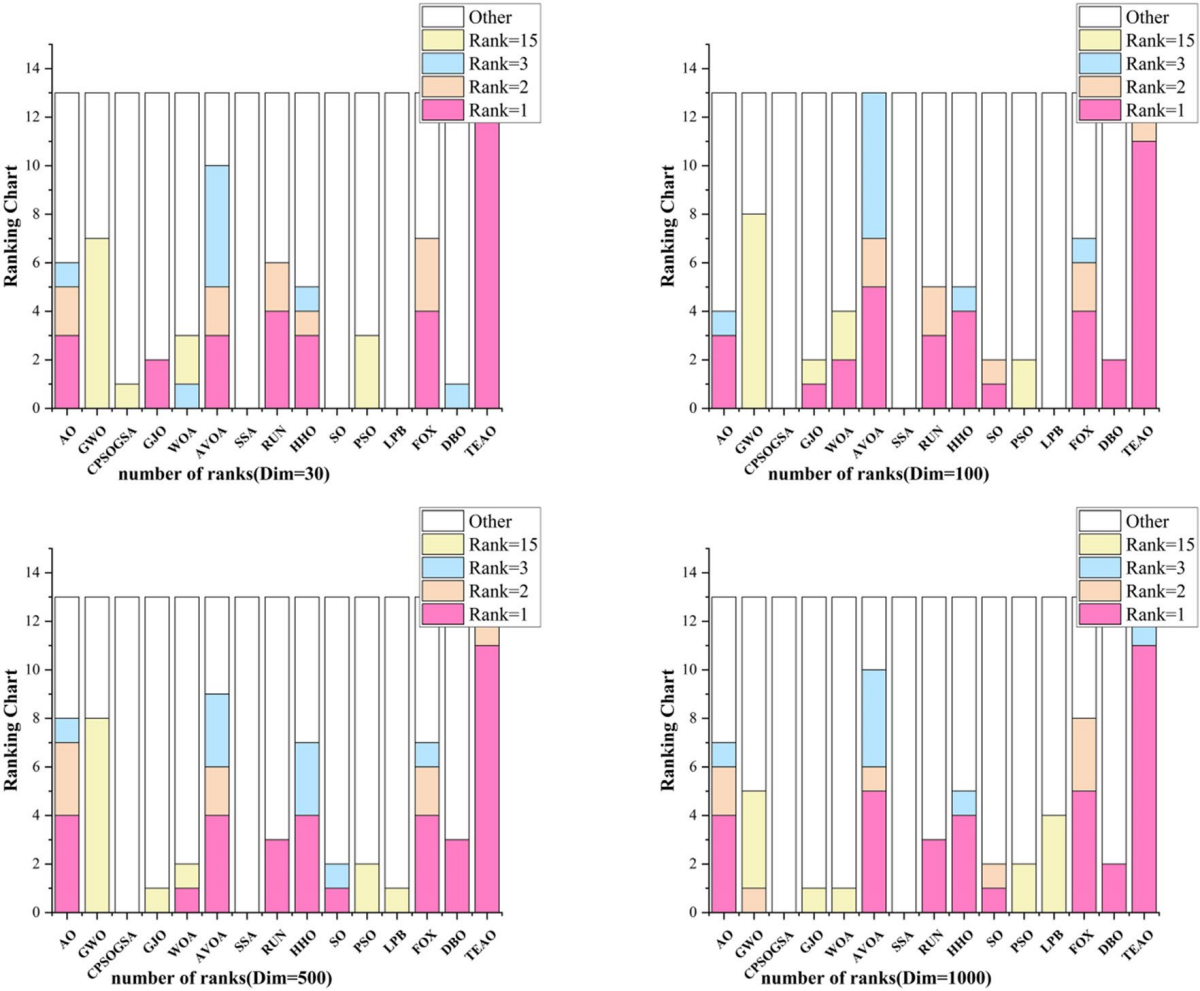
Test Function Ranking Statistics.

The chart in Fig. 10 illustrates the rankings of different algorithms across various dimensions. To better convey this information, we use a radar chart to depict the rankings of different algorithms on the test set. It is evident from the chart that the TEAO algorithm consistently maintains a leading position in the 30, 50, and 100 dimensions. In the 30-dimensional test, TEAO achieves the best average ranking across all 13 test functions, while AO only obtains 3. Furthermore, as the dimensionality increases, TEAO continues to exhibit robust optimization results. In the 100-dimensional test, TEAO maintains the best average ranking in 11 functions and the second-best ranking in 1 function. In the 500-dimensional test, TEAO similarly secures the best average ranking in 11 test functions and the second-best ranking in 1 function. In the 1000-dimensional test, TEAO attains the best average ranking in 11 test functions and the third-best ranking in 2 functions. It is noteworthy that, across these three dimensions, TEAO does not receive the worst ranking. Although TEAO does not achieve optimal results in F7 and F8 in the 100, 500, and 1000-dimensional tests, it still produces powerful results that are noticeably superior to most other algorithms in these scenarios.

From Tables 6, 7, 8, 9 and 10, it is evident that the averages (Ave) and standard deviations (Std) of TEAO are mostly presented in bold, indicating that TEAO consistently achieves the best experimental results across the majority of test sets. In contrast, AO and other comparative algorithms have only a few instances of bold data, further highlighting the robust performance of TEAO. Moreover, as the problem dimensions increase, TEAO continues to exhibit strong performance, while the performance of AO and other comparative algorithms deteriorates. The optimization capabilities of AO and other comparative algorithms noticeably decline with the increase in function dimensions, whereas TEAO is hardly affected. The experimental results demonstrate that our proposed chaotic initialization population strategy and new position update rules effectively balance exploration and exploitation, making the algorithm more conducive to solving complex high-dimensional problems. In summary, TEAO, as proposed, demonstrates good reliability and robustness, making it more competitive compared to other algorithms.

### Statistical test

In this section, we employ the Wilcoxon test and Friedman test to conduct a statistical analysis of the experimental results. The aim is to assess the differences between TEAO and other comparison algorithms in a rigorous manner.

#### Friedmann's test

By applying the non-parametric Friedman average rank test to evaluate and rank the experimental results of the SBOA algorithm and other algorithms on the 23 classical benchmark functions sets, we present the obtained rankings in Table 11. It is evident that SBOA consistently secures the top rank, highlighting the superior performance of our proposed optimizer compared to the other algorithms across the considered test sets.

**Table 11:** Friedman average rank sum test results.

Suites	The 23 classical benchmark functions							
Dimensions	30		100		500		1000	
Algorithms	Ave. Rank	Overall Rank	Ave. Rank	Overall Rank	Ave. Rank	Overall Rank	Ave. Rank	Overall Rank
AO	4.15	4	4.46	5	3.54	3	3.85	4
GWO	14.31	15	14.46	15	14.54	15	13.15	14
CPSOGSA	10.23	11	10.08	11	10.62	11	10.92	11
GJO	8.54	8	9.54	10	10.62	11	10.69	10
WOA	9.46	10	7.54	9	7.46	9	8.62	9
AVOA	3.15	2.5	2.08	2	2.69	2	2.69	2
SSA	11.73	13	12.69	14	12.00	12	11.77	12
RUN	3.15	2.5	3.77	3	4.15	5	4.00	6
HHO	4.38	5	4.00	4	3.62	4	4.00	6
SO	8.92	9	7.00	8	6.85	8	6.54	8
PSO	12.15	14	12.62	13	12.38	13	12.15	13
LPB	11.38	12	12.38	12	13.54	14	13.85	15
FOX	5.23	6	5.00	6	4.46	6	3.85	4
DBO	7.19	7	6.46	7	6.31	7	6.54	8
TEAO	1.00	1	1.38	1	1.38	1	1.31	1

#### Wilcoxon rank sum test

We conducted a Wilcoxon^67^ signed-rank sum test to compare the differences between TEAO and other competitive algorithms, and the results are reported in Tables 12, 13, 14, 15 and 16. When P < 0.05, it indicates a significant difference between TEAO and the competitive algorithm. Conversely, when P ≥ 0.05, there is no significant difference, and results without a significant difference are highlighted. 'NaN' indicates that the algorithm has obtained the global optimal solution.

**Table 12:** p-values of classical test functions (Dim = 30).

ID	AO	GWO	CPSOGSA	GJO	WOA	AVOA	SSA	RUN	HHO	SO	PSO	LPB	FOX	DBO
F1	1.21E-12	1.21E-12	1.21E-12	1.21E-12	1.21E-12	**0.333711**	1.21E-12	1.21E-12	1.21E-12	1.21E-12	1.21E-12	1.21E-12	**NaN**	1.21E-12
F2	1.21E-12	1.21E-12	1.21E-12	1.21E-12	1.21E-12	1.21E-12	1.21E-12	1.21E-12	1.21E-12	1.21E-12	1.21E-12	1.21E-12	1.21E-12	1.21E-12
F3	1.21E-12	1.21E-12	1.21E-12	1.21E-12	1.21E-12	1.21E-12	1.21E-12	1.21E-12	1.21E-12	1.21E-12	1.21E-12	1.21E-12	**NaN**	1.21E-12
F4	1.21E-12	1.21E-12	1.21E-12	1.21E-12	1.21E-12	1.21E-12	1.21E-12	1.21E-12	1.21E-12	1.21E-12	1.21E-12	1.21E-12	1.21E-12	1.21E-12
F5	9.51E-06	8.89E-10	2.92E-09	1.09E-10	4.08E-11	9.51E-06	6.07E-11	6.28E-06	9.51E-06	8.48E-09	3.02E-11	3.02E-11	2.80E-11	9.51E-06
F6	3.02E-11	6.69E-11	3.02E-11	3.02E-11	3.02E-11	3.02E-11	3.02E-11	3.02E-11	3.02E-11	3.02E-11	3.02E-11	3.02E-11	2.98E-11	3.02E-11
F7	**0.549327**	3.02E-11	3.02E-11	9.53E-07	3.82E-10	**0.684323**	3.02E-11	0.000812	**0.446419**	5.09E-06	3.02E-11	3.02E-11	**0.599689**	2.15E-10
F8	0.010741	7.03E-08	1.46E-07	2.98E-11	**0.185645**	0.001947	**0.09617**	**0.946941**	0.025061	**0.15786**	4.92E-05	**0.185645**	0.00905	**0.67341**
F9	**NaN**	1.21E-12	5.58E-10	**NaN**	**0.160802**	**NaN**	1.21E-12	**NaN**	**NaN**	1.31E-07	1.21E-12	1.21E-12	**NaN**	0.002788
F10	**NaN**	1.20E-12	7.29E-13	3.37E-13	4.23E-09	**NaN**	1.21E-12	**NaN**	**NaN**	1.69E-14	1.21E-12	1.21E-12	**NaN**	**0.333711**
F11	**NaN**	1.21E-12	0.011035	**NaN**	**0.081523**	**NaN**	1.21E-12	**NaN**	**NaN**	0.011035	1.21E-12	1.21E-12	**NaN**	**0.160802**
F12	3.02E-11	3.02E-11	3.02E-11	3.02E-11	3.02E-11	7.39E-11	3.02E-11	5.57E-10	3.02E-11	3.02E-11	3.02E-11	3.02E-11	3.02E-11	3.20E-09
F13	3.34E-11	3.02E-11	3.02E-11	3.02E-11	3.02E-11	5.07E-10	3.02E-11	2.37E-10	3.34E-11	3.02E-11	3.02E-11	3.02E-11	3.00E-11	4.08E-11

**Table 13:** p-values of classical test functions (Fixed dimension).

ID	AO	GWO	CPSOGSA	GJO	WOA	AVOA	SSA	RUN	HHO	SO	PSO	LPB	FOX	DBO
F14	7.14E-03	**9.52E-01**	9.24E-04	5.72E-05	6.53E-03	**0.109736**	**2.77E-01**	3.05E-03	4.22E-02	1.72E-03	**8.45E-01**	**1.03E-01**	1.88E-06	6.45E-06
F15	3.00E-11	3.28E-11	3.00E-11	3.00E-11	3.00E-11	3.00E-11	3.00E-11	4.94E-11	3.00E-11	3.00E-11	3.00E-11	3.00E-11	3.00E-11	3.00E-11
F16	1.45E-11	**4.41E-01**	1.45E-11	1.45E-11	3.02E-11	1.57E-04	1.45E-11	5.57E-10	6.16E-11	**4.47E-01**	**6.15E-02**	1.45E-11	1.45E-11	8.10E-03
F17	1.21E-12	**NaN**	1.21E-12	1.21E-12	1.21E-12	**NaN**	4.46E-12	1.94E-09	1.95E-09	**NaN**	**NaN**	1.21E-12	1.21E-12	**NaN**
F18	2.95E-11	6.57E-07	2.95E-11	2.95E-11	2.95E-11	2.95E-11	2.94E-11	1.23E-04	2.28E-09	6.50E-05	**3.06E-01**	2.95E-11	2.95E-11	5.63E-09
F19	1.67E-11	**2.44E-01**	1.67E-11	1.67E-11	1.67E-11	6.06E-07	1.66E-11	1.67E-11	1.67E-11	**1.48E-01**	4.08E-05	1.67E-11	1.67E-11	**1.34E-01**
F20	0.006669	8.48E-07	2.75E-03	3.83E-05	3.77E-04	0.000168	6.77E-05	0.000239	3.83E-05	2.03E-11	1.22E-02	**9.71E-01**	3.02E-11	**1.88E-01**
F21	3.02E-11	5.23E-04	3.02E-11	3.02E-11	3.02E-11	7.44E-09	**0.411904**	**0.958731**	3.02E-11	**0.765292**	**3.40E-01**	3.02E-11	3.02E-11	**0.170777**
F22	2.95E-11	3.76E-02	2.95E-11	2.95E-11	2.95E-11	0.000245	**7.17E-01**	**0.171242**	2.95E-11	**1.39E-01**	**3.25E-01**	2.95E-11	2.95E-11	0.036544
F23	3.02E-11	2.59E-03	3.02E-11	3.02E-11	3.02E-11	1.91E-08	**5.30E-01**	**0.56922**	3.02E-11	**3.96E-01**	2.51E-05	6.70E-11	3.02E-11	**0.097923**

**Table 14:** p-values of classical test functions (Dim = 100).

ID	AO	GWO	CPSOGSA	GJO	WOA	AVOA	SSA	RUN	HHO	SO	PSO	LPB	FOX	DBO
F1	1.21E-12	1.21E-12	1.21E-12	1.21E-12	1.21E-12	**NaN**	1.21E-12	1.21E-12	1.21E-12	1.21E-12	1.21E-12	1.21E-12	**NaN**	1.21E-12
F2	1.21E-12	1.21E-12	1.21E-12	1.21E-12	1.21E-12	1.21E-12	1.21E-12	1.21E-12	1.21E-12	1.21E-12	1.21E-12	1.21E-12	1.21E-12	1.21E-12
F3	1.21E-12	1.21E-12	1.21E-12	1.21E-12	1.21E-12	1.21E-12	1.21E-12	1.21E-12	1.21E-12	1.21E-12	1.21E-12	1.21E-12	**NaN**	1.21E-12
F4	1.21E-12	1.21E-12	1.21E-12	1.21E-12	1.21E-12	1.21E-12	1.21E-12	1.21E-12	1.21E-12	1.21E-12	1.21E-12	1.21E-12	1.21E-12	1.21E-12
F5	7.96E-03	3.02E-11	2.44E-09	5.49E-11	3.02E-11	7.96E-03	3.02E-11	3.37E-04	7.96E-03	1.17E-05	3.02E-11	3.02E-11	2.26E-11	3.56E-04
F6	3.02E-11	3.02E-11	3.02E-11	3.02E-11	3.02E-11	3.02E-11	3.02E-11	3.02E-11	3.02E-11	3.02E-11	3.02E-11	3.02E-11	2.95E-11	3.02E-11
F7	**0.067869**	3.02E-11	3.02E-11	3.82E-10	2.23E-09	**0.923442**	3.02E-11	0.003501	**0.923442**	**9.33E-02**	3.02E-11	3.02E-11	**0.946956**	2.15E-10
F8	3.49E-09	6.71E-10	1.07E-09	2.60E-10	0.026069	0.009879	**0.662717**	**0.864986**	0.007614	0.022353	**1.86E-01**	0.033864	**0.958729**	**0.437616**
F9	**NaN**	1.21E-12	1.21E-12	**0.333711**	**NaN**	**NaN**	1.21E-12	**NaN**	**NaN**	5.85E-09	1.21E-12	1.21E-12	**NaN**	**0.333711**
F10	**NaN**	1.21E-12	1.21E-12	7.98E-13	2.85E-07	**NaN**	1.21E-12	**NaN**	**NaN**	1.69E-14	1.21E-12	1.21E-12	2.36E-05	**NaN**
F11	**NaN**	1.21E-12	1.2E-12	**NaN**	**NaN**	**NaN**	1.21E-12	**NaN**	**NaN**	**NaN**	1.21E-12	1.21E-12	**NaN**	**NaN**
F12	3.69E-11	3.02E-11	3.02E-11	3.02E-11	3.02E-11	3.02E-11	3.02E-11	3.02E-11	3.02E-11	3.02E-11	3.02E-11	3.02E-11	3.01E-11	3.02E-11
F13	3.02E-11	3.02E-11	3.02E-11	3.02E-11	3.02E-11	3.02E-11	3.02E-11	3.02E-11	3.02E-11	3.02E-11	3.02E-11	3.02E-11	3.02E-11	3.02E-11

**Table 15:** p-values of classical test functions (Dim = 500).

ID	AO	GWO	CPSOGSA	GJO	WOA	AVOA	SSA	RUN	HHO	SO	PSO	LPB	FOX	DBO
F1	1.21E-12	1.21E-12	1.21E-12	1.21E-12	1.21E-12	**NaN**	1.21E-12	1.21E-12	1.21E-12	1.21E-12	1.21E-12	1.21E-12	**NaN**	1.21E-12
F2	1.21E-12	1.21E-12	1.21E-12	1.21E-12	1.21E-12	1.21E-12	1.21E-12	1.21E-12	1.21E-12	1.21E-12	1.21E-12	1.21E-12	1.21E-12	1.21E-12
F3	1.21E-12	1.21E-12	1.21E-12	1.21E-12	1.21E-12	1.21E-12	1.21E-12	1.21E-12	1.21E-12	1.21E-12	1.21E-12	1.21E-12	**NaN**	1.21E-12
F4	1.21E-12	1.21E-12	1.21E-12	1.21E-12	1.21E-12	1.21E-12	1.21E-12	1.21E-12	1.21E-12	1.21E-12	1.21E-12	1.21E-12	1.21E-12	1.21E-12
F5	**1.86E-01**	3.02E-11	3.02E-11	3.02E-11	3.02E-11	**1.86E-01**	3.02E-11	5.56E-04	**1.86E-01**	6.28E-06	3.02E-11	3.02E-11	2.63E-11	3.02E-11
F6	3.02E-11	3.02E-11	3.02E-11	3.02E-11	3.02E-11	3.02E-11	3.02E-11	3.02E-11	3.02E-11	3.02E-11	3.02E-11	3.02E-11	3.01E-11	3.02E-11
F7	**0.05012**	3.02E-11	3.02E-11	3.02E-11	1.55E-09	**0.340288**	3.02E-11	0.00077	**0.970516**	4.06E-02	3.02E-11	3.02E-11	**0.428963**	1.41E-09
F8	4.57E-05	7.91E-03	3.64E-03	6.17E-06	**0.10518**	**0.074585**	0.029071	**0.728047**	0.004194	**0.06764**	**3.87E-01**	**0.347426**	**0.500786**	**0.370678**
F9	**NaN**	1.21E-12	1.21E-12	8.88E-13	**0.333711**	**NaN**	1.21E-12	**NaN**	**NaN**	4.19E-02	1.21E-12	1.21E-12	**NaN**	**NaN**
F10	**NaN**	1.21E-12	1.21E-12	1.21E-12	2.90E-08	**NaN**	1.21E-12	**NaN**	**NaN**	2.71E-14	1.21E-12	1.21E-12	1.97E-11	**NaN**
F11	**NaN**	1.21E-12	1.21E-12	1.21E-12	**NaN**	**NaN**	1.21E-12	**NaN**	**NaN**	**NaN**	1.21E-12	1.21E-12	**NaN**	**NaN**
F12	3.02E-11	3.02E-11	3.02E-11	3.02E-11	3.02E-11	3.02E-11	3.02E-11	3.02E-11	3.02E-11	3.02E-11	3.02E-11	3.02E-11	2.95E-11	3.02E-11
F13	3.02E-11	3.02E-11	3.02E-11	3.02E-11	3.02E-11	3.02E-11	3.02E-11	3.02E-11	3.02E-11	3.02E-11	3.02E-11	3.02E-11	3.02E-11	3.02E-11

**Table 16:** p-values of classical test functions (Dim = 1000).

ID	AO	GWO	CPSOGSA	GJO	WOA	AVOA	SSA	RUN	HHO	SO	PSO	LPB	FOX	DBO
F1	1.21E-12	1.21E-12	1.21E-12	1.21E-12	1.21E-12	**NaN**	1.21E-12	1.21E-12	1.21E-12	1.21E-12	1.21E-12	1.21E-12	**NaN**	1.21E-12
F2	1.21E-12	1.69E-14	1.21E-12	1.21E-12	1.21E-12	**NaN**	1.21E-12	1.21E-12	1.21E-12	1.21E-12	1.21E-12	1.21E-12	**NaN**	1.21E-12
F3	1.21E-12	1.21E-12	1.21E-12	1.21E-12	1.21E-12	1.21E-12	1.21E-12	1.21E-12	1.21E-12	1.21E-12	1.21E-12	1.21E-12	**NaN**	1.21E-12
F4	1.21E-12	1.21E-12	1.21E-12	1.21E-12	1.21E-12	1.21E-12	1.21E-12	1.21E-12	1.21E-12	1.21E-12	1.21E-12	1.21E-12	1.21E-12	1.21E-12
F5	1.07E-07	3.02E-11	3.02E-11	3.02E-11	3.02E-11	1.07E-07	3.02E-11	3.16E-10	1.07E-07	5.57E-10	3.02E-11	3.02E-11	2.26E-11	3.02E-11
F6	3.02E-11	3.02E-11	3.02E-11	3.02E-11	3.02E-11	3.02E-11	3.02E-11	3.02E-11	3.02E-11	3.02E-11	3.02E-11	3.02E-11	2.63E-11	3.02E-11
F7	0.011228	3.02E-11	3.02E-11	3.02E-11	4.62E-10	**0.559231**	3.02E-11	1.17E-05	**0.520145**	**1.49E-01**	3.02E-11	3.02E-11	**0.403538**	1.55E-09
F8	2.62E-08	1.53E-06	**2.75E-01**	5.63E-06	0.001851	0.00176	**0.160239**	0.006395	0.00055	0.001435	9.93E-03	0.007645	**0.100584**	0.001946
F9	**NaN**	1.21E-12	1.21E-12	1.18E-12	**0.160742**	**NaN**	1.21E-12	**NaN**	**NaN**	**8.15E-02**	1.21E-12	1.21E-12	**NaN**	**NaN**
F10	**NaN**	1.21E-12	1.21E-12	1.21E-12	1.22E-08	**NaN**	1.21E-12	**NaN**	**NaN**	1.97E-13	1.21E-12	1.21E-12	1.17E-13	**0.333711**
F11	**NaN**	1.21E-12	1.21E-12	1.21E-12	**0.333711**	**NaN**	1.21E-12	**NaN**	**NaN**	**NaN**	1.21E-12	1.21E-12	**NaN**	**NaN**
F12	3.02E-11	3.02E-11	3.02E-11	3.02E-11	3.02E-11	3.02E-11	3.02E-11	3.02E-11	3.02E-11	3.02E-11	3.02E-11	3.02E-11	2.92E-11	3.02E-11
F13	3.02E-11	3.02E-11	3.02E-11	3.02E-11	3.02E-11	3.02E-11	3.02E-11	3.02E-11	3.02E-11	3.02E-11	3.02E-11	3.02E-11	3.00E-11	3.02E-11

The results reveal that as the complexity of the dimension or test functions increases, the distinction between TEAO and other algorithms becomes more prominent. This suggests that TEAO stands out as a unique algorithm with the most outstanding comprehensive performance compared to its competitors.

## TEAO is used for the engineering design problems

The previous experimental results in section "Experimental results and analysis" demonstrate that TEAO significantly enhances overall performance when compared to the original AO. This section aims to further verify the efficacy of TEAO by applying it to solve six classic engineering problems (three-bar truss design, pressure vessel, tension/compression spring design, welded beam design and gear train design). The results will be compared those of GWO, CPSOGSA, GJO, WOA, AVOA, SSA, RUN, HHO, SO, PSO, LPB, FOX, DBO and AO to evaluate TEAO's performance.

### Three-bar truss design (TBTD)

**Figure 11:**
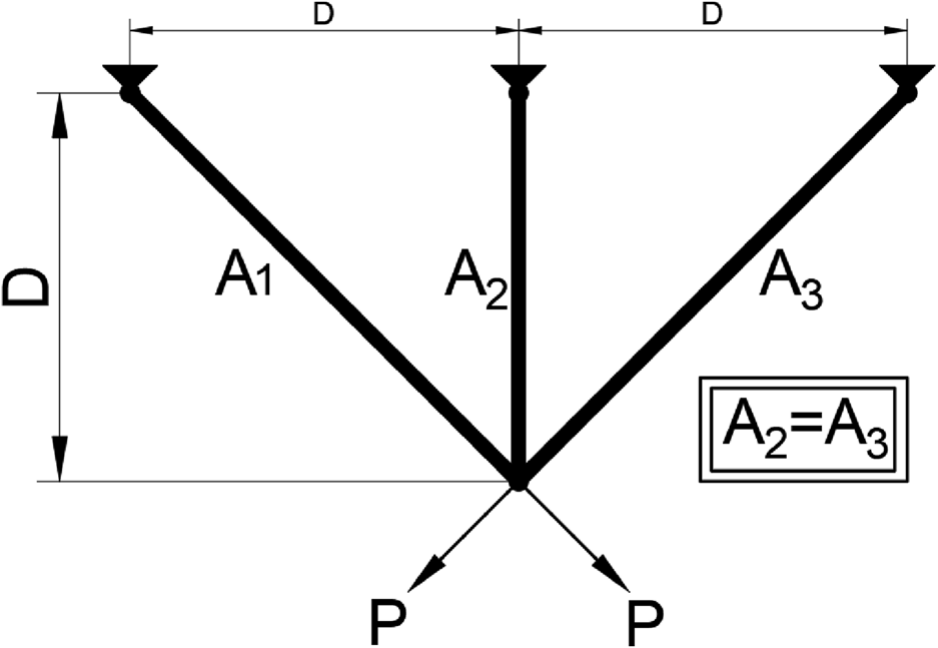
Schematic diagram of the three-bar truss structure.

The objective of the three-bar truss design problem is to minimize the overall structural weight by controlling two parameter variables, which originates from the field of civil engineering. The structure is depicted in Fig. 11 and Eq. (27) describes the mathematical model for this problem.

27 $$\begin{aligned} {\text{Consider}}: & \quad \vec{x} = \left[ {x_{1} x_{2} } \right] = \left[ {A_{1} A_{2} } \right], \\ {\text{Minimize}}: & \quad f\left( {\vec{x}} \right) = l*\left( {2\sqrt {2x_{1} } + x_{2} } \right), \\ {\text{Subject to}}: & \quad g_{1} \left( {\vec{x}} \right) = \frac{{\sqrt {2x_{1} } + x_{2} }}{{\sqrt {2x_{1}^{2} } + 2x_{1} x_{2} }}P - \sigma \le 0, \\ & \quad g_{2} \left( {\vec{x}} \right) = \frac{{x_{2} }}{{\sqrt {2x_{1}^{2} } + 2x_{1} x_{2} }}P - \sigma \le 0, \\ & \quad g_{3} \left( {\vec{x}} \right) = \frac{1}{{\sqrt {2x_{2} } + x_{1} }}P - \sigma \le 0, \\ {\text{Parameter range}}: & \quad 0 \le x_{1} ,x_{2} \le 1, \\ \end{aligned}$$

where $$l = 100\;{\text{cm}},P = 2\;{\text{KN}}/{\text{cm}}^{2} ,\sigma = 2\;{\text{KN}}/{\text{cm}}^{2} .$$

Table 17 presents the optimization results of TEAO compared with 14 other algorithms for the three-bar truss design problem. It is evident from the table that TEAO simultaneously achieves the optimal cost of 2.64E + 02. These results demonstrate that TEAO outperforms other comparison algorithms in solving the three-bar truss design problem.

**Table 17:** Comparison results for the three-bar truss design.

Algorithm	Optimal values for Variable		Optimal value	Ranking
	***A***_***1***_	***A***_***2***_		
AO	7.81E-01	4.34E-01	2.64E + 02	13
GWO	7.90E-01	4.04E-01	2.64E + 02	8
CPSOGSA	7.97E-01	4.02E-01	2.66E + 02	14
GJO	7.86E-01	4.17E-01	2.64E + 02	9
WOA	7.96E-01	3.88E-01	2.64E + 02	10
AVOA	7.89E-01	4.06E-01	2.64E + 02	4
SSA	7.90E-01	4.06E-01	2.64E + 02	5
RUN	7.90E-01	4.05E-01	2.64E + 02	6
HHO	8.01E-01	3.75E-01	2.64E + 02	11
SO	7.89E-01	4.07E-01	2.64E + 02	3
PSO	7.89E-01	4.07E-01	2.64E + 02	2
LPB	8.60E-01	2.37E-01	2.67E + 02	15
FOX	7.85E-01	4.19E-01	2.64E + 02	12
DBO	7.87E-01	4.12E-01	2.64E + 02	7
**TEAO**	**7.89E-01**	**4.08E-01**	**2.64E + 02**	**1**

### Pressure vessel design (PVD)

**Figure 12:**
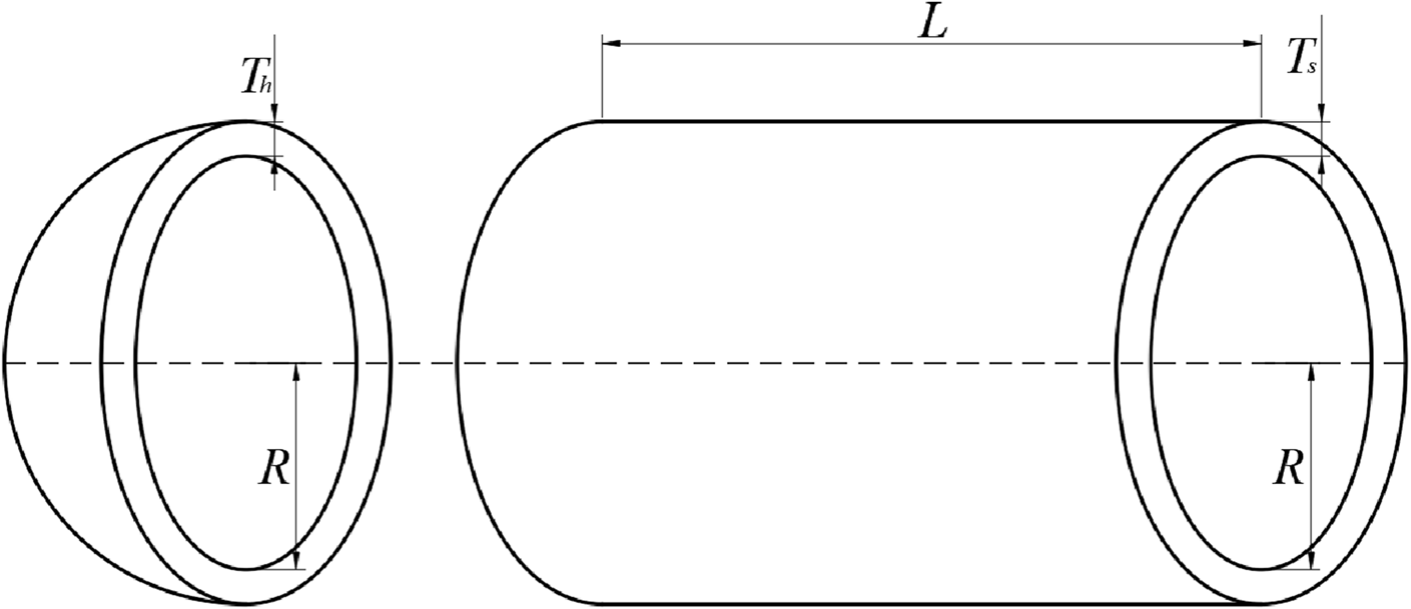
Schematic diagram of pressure vessel structure.

The structure of the pressure vessel design problem is depicted in Fig. 12. The objective of this design problem is to minimize the cost while fulfilling usage requirements. The four optimization parameters comprise vessel thickness (*T*_*S*_), head thickness (*T*_*h*_), inner radius (*R*) and head length (*L*). The mathematical model for this problem is represented by Eq. (28).

28 $$\begin{aligned} {\text{Consider}}: & \quad \vec{x} = \left[ {x_{1} x_{2} x_{3} x_{4} } \right] = \left[ {T_{{\text{s}}} T_{h} { }R L} \right], \\ {\text{Minimize}}: & \quad f\left( {\vec{x}} \right) = 0.6224x_{1} x_{3} x_{4} + 1.7781x_{2} x_{3}^{2} + 3.1661x_{1}^{2} x_{4} + 19.84x_{1}^{2} x_{3} , \\ {\text{Subject to}}: & \quad g_{1} \left( {\vec{x}} \right) = - x_{1} + 0.0193x_{3} \le 0, \\ & \quad g_{2} \left( {\vec{x}} \right) = - x_{3} + 0.00954x_{3} \le 0, \\ & \quad g_{3} \left( {\vec{x}} \right) = - \pi x_{3}^{2} x_{4} - \frac{4}{3}\pi x_{3}^{3} + 1296000 \le 0, \\ & \quad g_{4} \left( {\vec{x}} \right) = x_{4} - 240 \le 0, \\ {\text{Parameters range}}: & \quad 0 \le x_{1} ,x_{2} \le 99,{ }10 \le x_{3} ,x_{4} \le 200. \\ \end{aligned}$$

As evident from the results in Table. 18, TEAO outperforms all other comparison algorithms, achieving a minimum cost of 5.89E + 03. This indicates the superiority of TEAO in addressing this engineering problem.

**Table 18:** Comparison results for the pressure vessel design problem.

Algorithm	Optimal values for Variable				Optimal value	Ranking
	***T***_***s***_	***T***_***h***_	***R***	***L***		
AO	9.77E-01	4.81E-01	4.95E + 01	1.03E + 02	6.46E + 03	9
GWO	9.29E-01	4.61E-01	4.81E + 01	1.14E + 02	6.20E + 03	7
CPSOGSA	7.82E-01	3.87E-01	4.05E + 01	1.97E + 02	5.89E + 03	1
GJO	8.50E-01	4.24E-01	4.39E + 01	1.56E + 02	5.90E + 03	3
WOA	1.41E + 00	8.04E-01	6.52E + 01	1.00E + 01	9.30E + 03	14
AVOA	8.73E-01	4.32E-01	4.53E + 01	1.41E + 02	6.07E + 03	6
SSA	1.06E + 00	5.24E-01	5.49E + 01	8.02E + 01	7.22E + 03	10
RUN	1.26E + 00	6.22E-01	6.52E + 01	1.00E + 01	7.32E + 03	12
HHO	1.25E + 00	6.45E-01	6.47E + 01	1.22E + 01	7.49E + 03	13
SO	8.09E-01	4.10E-01	4.17E + 01	1.82E + 02	6.00E + 03	4
PSO	9.58E-01	4.74E-01	4.96E + 01	1.01E + 02	6.27E + 03	8
LPB	8.25E-01	4.15E-01	4.23E + 01	1.75E + 02	6.06E + 03	5
FOX	1.50E + 00	7.09E-01	6.92E + 01	1.00E + 01	9.81E + 03	15
DBO	1.26E + 00	6.22E-01	6.52E + 01	1.00E + 01	7.32E + 03	11
**TEAO**	**7.85E-01**	**3.88E-01**	**4.07E + 01**	**1.95E + 02**	**5.89E + 03**	**1**

## Tension/compression spring design (T/CSD)

**Figure 13:**
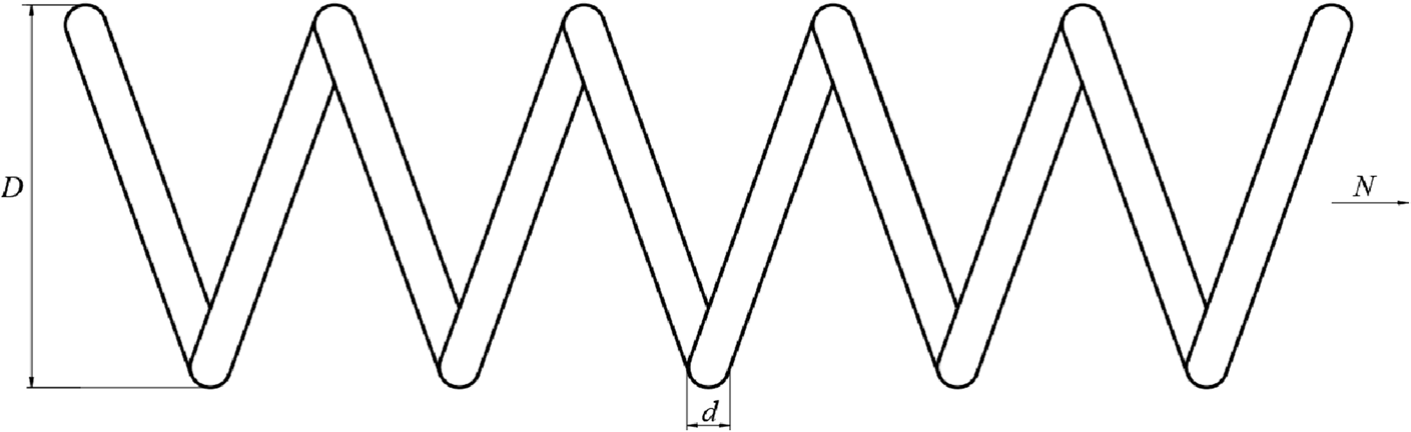
Tension/compression spring structure diagram.

The design objective is to determine three crucial parameters of the spring by minimizing the weight of the tension/compression spring, namely wire diameter (*d*), coil diameter (*D*) and coil number (*N*). The structure of the engineering problem is illustrated in Fig. 13, and the corresponding mathematical model is presented in Eq. (29).

29 $$\begin{aligned} {\text{Consider}}: & \quad \vec{x} = \left[ {\begin{array}{*{20}l} {x_{1} } \hfill & {x_{2} } \hfill & {x_{3} } \hfill \\ \end{array} } \right] = \left[ {d D N} \right]{,} \\ {\text{Minimize}}: & \quad f\left( {\vec{x}} \right) = \left( {x_{3} + 2} \right)x_{2} x_{1}^{2} , \\ {\text{Subject to}}: & \quad g_{1} \left( {\vec{x}} \right) = 1 - \frac{{x_{2}^{3} x_{3} }}{{71785x_{1}^{4} }} \le 0, \\ & \quad g_{2} \left( {\vec{x}} \right) = \frac{{4x_{2}^{2} - x_{1} x_{2} }}{{12566\left( {x_{2} x_{1}^{3} - x_{1}^{4} } \right)}} + \frac{1}{{5108x_{1}^{2} }} \le 0, \\ & \quad g_{3} \left( {\vec{x}} \right) = 1 - \frac{{140.45x_{1} }}{{x_{2}^{2} x_{3} }} \le 0, \\ & \quad g_{4} \left( {\vec{x}} \right) = \frac{{x_{1} + x_{2} }}{1.5} - 1 \le 0 \\ {\text{Parameters range}}: & \quad 0.05 \le x_{1} \le 2, 0.25 \le x_{2} \le 1.3, 2 \le x_{3} \le 15. \\ \end{aligned}$$

Table 19 presents the optimization results of TEAO and 14 other diverse comparison algorithms for tension/compression spring design. The table reveals that TEAO outperforms other comparison algorithms, yielding an optimal value of 1.27E-02.

**Table 19:** Comparison results for the tension/compression spring design problem.

Algorithm	Optimal values for Variable			Optimal value	Ranking
	***d***	***D***	***N***		
AO	6.48E-02	7.48E-01	3.24E + 00	1.65E-02	15
GWO	5.75E-02	5.14E-01	5.79E + 00	1.32E-02	11
CPSOGSA	5.31E-02	3.92E-01	9.49E + 00	1.27E-02	2
GJO	5.16E-02	3.54E-01	1.15E + 01	1.27E-02	6
WOA	5.86E-02	5.46E-01	5.20E + 00	1.35E-02	13
AVOA	5.43E-02	4.24E-01	8.21E + 00	1.28E-02	7
SSA	5.00E-02	3.12E-01	1.48E + 01	1.31E-02	8
RUN	5.00E-02	3.17E-01	1.40E + 01	1.27E-02	4
HHO	6.24E-02	6.72E-01	3.57E + 00	1.46E-02	14
SO	5.36E-02	4.06E-01	8.91E + 00	1.27E-02	5
PSO	5.79E-02	5.26E-01	5.56E + 00	1.33E-02	12
LPB	5.69E-02	4.95E-01	6.22E + 00	1.32E-02	10
FOX	5.00E-02	3.14E-01	1.47E + 01	1.31E-02	9
DBO	5.00E-02	3.17E-01	1.40E + 01	1.27E-02	3
**TEAO**	**5.24E-02**	**3.74E-01**	**1.04E + 01**	**1.27E-02**	**1**

### Welded beam design (WBD)

**Figure 14:**
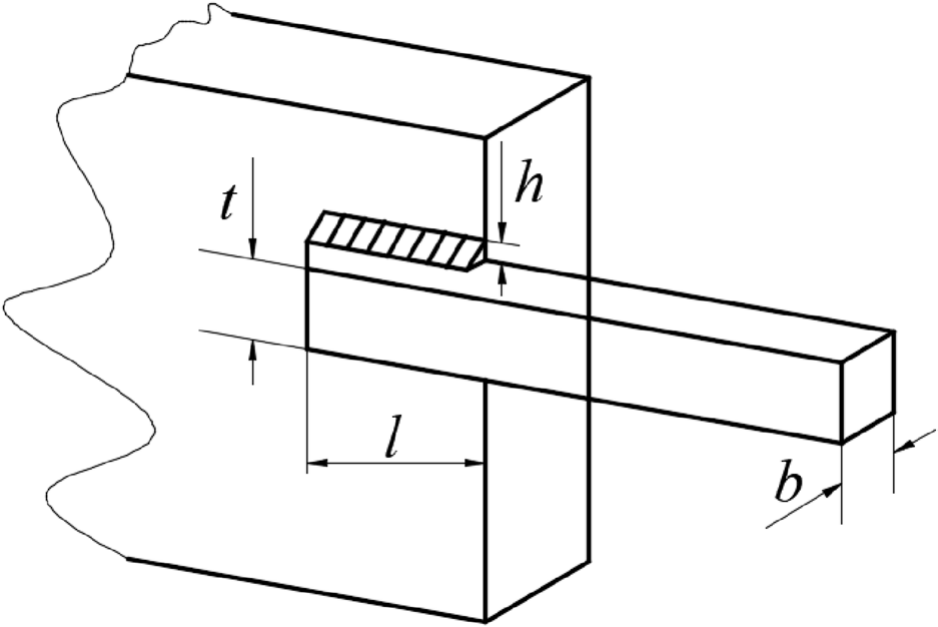
Welding beam structure diagram.

The design of a welded beam represents a typical nonlinear programming problem. It aims to minimize the manufacturing cost of welded beams while constraining parameters such as Thickness (*h*), Length (*l*), Height (*t*), Thickness (*b*) and weld of the beam reinforcement. The structure of the optimization problem is depicted in Fig. 14, and its mathematical model is presented in Eq. (30).

30 $$\begin{aligned} {\text{Consider}}: & \quad \vec{x} = \left[ {x_{1} x_{2} x_{3} x_{4} \left] = \right[\begin{array}{*{20}c} h & l & t & b \\ \end{array} } \right], \\ {\text{Minimize}}: & \quad f\left( {\vec{x}} \right) = f\left( {\vec{x}} \right) = 1.10471x_{1}^{2} x_{2} + 0.04811x_{3} x_{4} \left( {14.0 + x_{2} } \right), \\ {\text{Subject to}}: & \quad g_{1} \left( {\vec{x}} \right) = \tau \left( {\vec{x}} \right) - \tau_{max} < 0, \\ & \quad g_{2} \left( {\vec{x}} \right) = \sigma \left( {\vec{x}} \right) - \sigma_{max} < 0, \\ & \quad g_{3} \left( {\vec{x}} \right) = \delta \left( {\vec{x}} \right) - \delta_{max} < 0, \\ & \quad g_{4} \left( {\vec{x}} \right) = x_{1} - x_{4} < 0 \\ & \quad g_{5} \left( {\vec{x}} \right) = P - P_{c} \left( {\vec{x}} \right) < 0, \\ & \quad g_{6} \left( {\vec{x}} \right) = 0.125 - x_{1} < 0, \\ & \quad g_{7} \left( {\vec{x}} \right) = 1.10471x_{1}^{2} + 0.04811x_{3} x_{4} \left( {14.0 + x_{2} } \right) - 5.0 < 0, \\ {\text{Parameters range}}: & \quad 0.1 < x_{1} ,x_{4} < 2,0.1 < x_{2} ,x_{3} \le 10, \\ {\text{where}}, & \quad \tau \left( {\vec{x}} \right) = \sqrt {\left( {\tau^{\prime}} \right)^{2} + 2\tau^{\prime}\tau^{\prime\prime}\frac{{x_{2} }}{2R} + \left( {\tau^{\prime}} \right)^{2} } , \\ & \quad \tau^{\prime} = \frac{p}{{\sqrt {2x_{1} x_{2} } }},\quad \tau^{\prime\prime} = \frac{MR}{1}, \\ & \quad M = P\left( {L + \frac{{x_{2} }}{2}} \right), \\ & \quad R = \sqrt {\frac{{x_{2}^{2} }}{4} + \left( {\frac{{x_{1} + x_{2} }}{2}} \right)^{2} } , \\ & \quad J = 2\left\{ {\sqrt {2x_{1} x_{2} } \left[ {\frac{{x_{2}^{2} }}{4} + \left( {\frac{{x_{1} + x_{2} }}{2}} \right)^{2} } \right]} \right\}, \\ & \quad \sigma \left( {\vec{x}} \right) = \frac{6PL}{{x_{4} x_{2}^{2} }},\delta \left( {\vec{x}} \right) = \frac{{6PL^{2} }}{{Ex_{2}^{2} x_{4} }}, \\ & \quad P_{c} \left( {\overline{x}} \right) = \frac{{\sqrt {\frac{40x}{{36}}} }}{{L^{2} }}\left( {1 - \frac{{x_{2}^{2} }}{2L}\sqrt{\frac{E}{4C}} } \right), \\ & \quad P = 6000Lb,L = 14{\text{in}},\delta_{max} = 0.25{\text{in}}, \\ & \quad E = 30 \times 1^{6} psi,G = 12 \times 10^{6} psi, \\ & \quad \tau_{max} = 13600psi,\sigma_{max} = 30000{\text{psi}}. \\ \end{aligned}$$

Table 20 presents the optimization results of various competitive algorithms for welded beam design problems. The results indicate that TEAO outperforms other algorithms significantly, with an optimal value of 1.67E + 00.

**Table 20:** Comparison results for the welded beam problem.

Algorithm	Optimal values for Variable				Optimal value	Ranking
	***h***	***l***	***t***	***b***		
AO	1.25E-01	6.06E + 00	8.68E + 00	2.23E-01	1.97E + 00	14
GWO	1.99E-01	3.35E + 00	9.19E + 00	1.99E-01	1.67E + 00	2
CPSOGSA	2.47E-01	2.95E + 00	7.84E + 00	2.73E-01	1.95E + 00	13
GJO	1.85E-01	3.65E + 00	9.19E + 00	1.99E-01	1.69E + 00	4
WOA	4.57E-01	2.14E + 00	4.94E + 00	6.87E-01	3.13E + 00	15
AVOA	1.94E-01	3.43E + 00	9.19E + 00	1.99E-01	1.68E + 00	3
SSA	1.33E-01	5.21E + 00	9.19E + 00	1.99E-01	1.79E + 00	6
RUN	1.25E-01	5.57E + 00	9.19E + 00	1.99E-01	1.82E + 00	11
HHO	2.19E-01	3.50E + 00	8.70E + 00	2.22E-01	1.81E + 00	8
SO	2.07E-01	3.60E + 00	8.54E + 00	2.38E-01	1.89E + 00	12
PSO	1.94E-01	3.43E + 00	9.19E + 00	1.99E-01	1.80E + 00	7
LPB	2.33E-01	2.99E + 00	8.41E + 00	2.38E-01	1.81E + 00	9
FOX	1.85E-01	3.49E + 00	1.00E + 01	1.96E-01	1.78E + 00	5
DBO	1.25E-01	5.57E + 00	9.19E + 00	1.99E-01	1.82E + 00	10
**TEAO**	**1.99E-01**	**3.34E + 00**	**9.19E + 00**	**1.99E-01**	**1.67E + 00**	**1**

### Rolling element bearing design (REBD)

**Figure 15:**
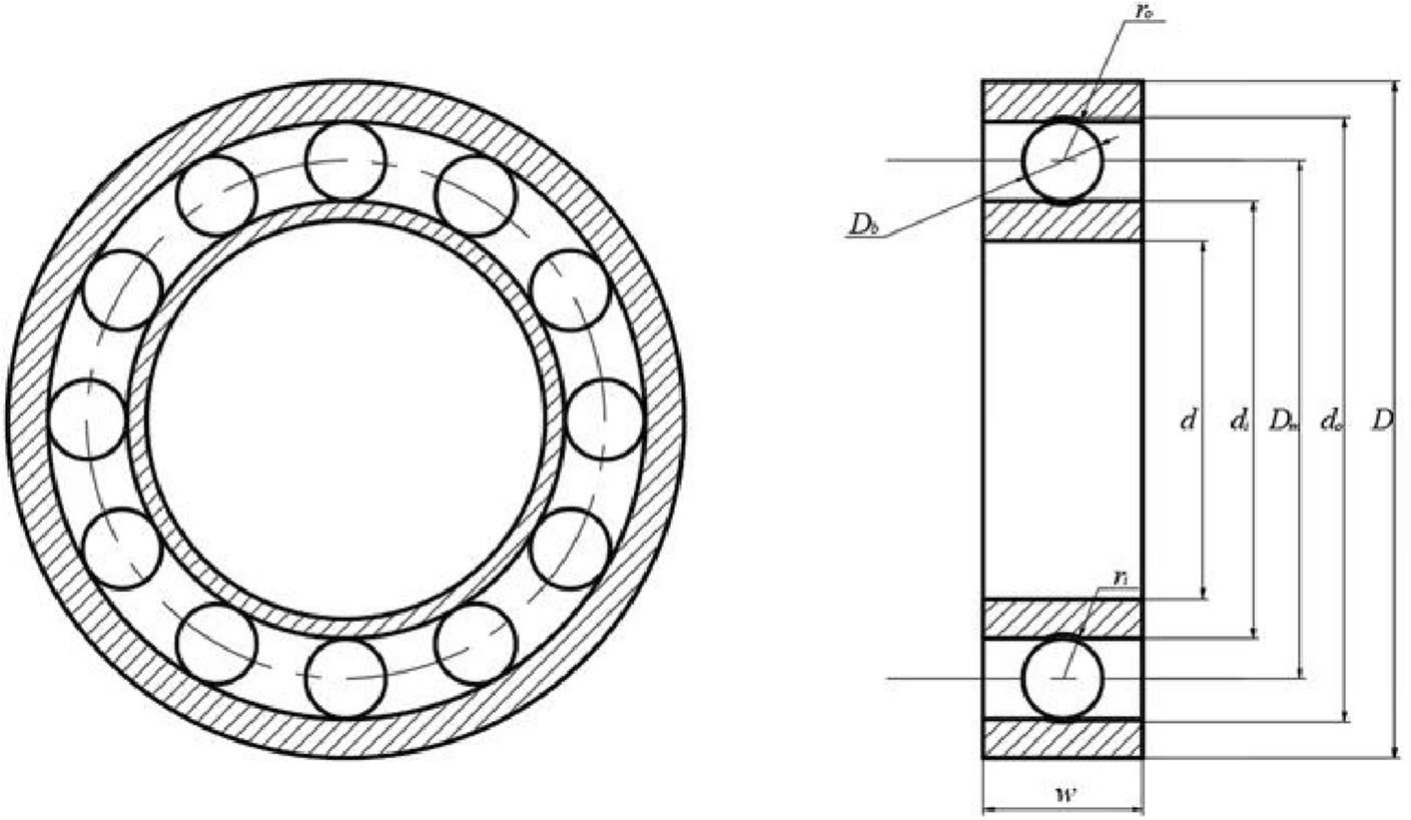
Rolling bearing structure diagram.

The design of rolling element bearings presents a complex nonlinear challenge. The bearing's ability to support loads is restricted by ten parameters, encompassing five design variables: pitch diameter $({D}_{m})$, ball diameter $({D}_{b})$, and curvature coefficients of the outer and inner raceways ($({f}_{o})$ and $({f}_{c})$), as well as the total number of balls $(Z)$. The remaining five design parameters, including $e$, $\epsilon$, $\zeta$, $({KD}_{max})$, and $({KD}_{min})$, are used solely in the constraints. To better illustrate the structure of the optimization problem, Fig. 15 is provided. Additionally, the mathematical model of the problem can be found in Eq. (31).

31 $$\begin{aligned} {\text{Consider}}: & \quad \vec{x} = \left[ {\begin{array}{*{20}c} {x_{1} } & {x_{2} } & {x_{3} } & {x_{4} } & {x_{5} } & {x_{6} } & {x_{7} } & {x_{8} } & {x_{9} } & {x_{10} } \\ \end{array} \left] = \right[\begin{array}{*{20}c} {D_{m} } & {D_{b} } & {f_{o} } & {f_{i} } & Z & {e \zeta } & {KD_{max} } & {KD_{min} } \\ \end{array} } \right] \\ {\text{Minimize}}: & \quad f\left( {\overline{x}} \right) = \left\{ {\begin{array}{*{20}c} {f_{c} Z^{2/3} D_{b}^{1.8} ,if D_{b} \le 25.4mm} \\ {3.647f_{c} Z^{2/3} D_{b}^{1.4} , otherwise} \\ \end{array} } \right. \\ {\text{Subject to}}: & \quad g_{1} \left( {\overline{x}} \right) = Z - \frac{{\phi_{0} }}{{2{\text{sin}}^{ - 1} \left( {D_{b} /D_{m} } \right)}} - 1 \le 0{,} \\ & \quad g_{2} \left( {\overline{x}} \right) = K_{Dmin} \left( {D - d} \right) - 2D_{b} \le 0{,} \\ & \quad g_{3} \left( {\overline{x}} \right) = 2D_{b} - K_{Dmax} \left( {D - d} \right) \le 0{,} \\ & \quad g_{4} \left( {\overline{x}} \right) = D_{b} - B_{w} \le 0 \\ & \quad g_{5} \left( {\overline{x}} \right) = 0.5\left( {D + d} \right) - D_{m} \le 0{,} \\ & \quad g_{6} \left( {\overline{x}} \right) = D_{m} - \left( {0.5 + e} \right)\left( {D + d} \right) \le 0{,} \\ & \quad g_{7} \left( {\overline{x}} \right) = D_{b} - 0.5\left( {D - D_{m} - D_{b} } \right) \le 0{,} \\ & \quad g_{8} \left( {\overline{x}} \right) = 0.515 - f_{i} \le 0{,} \\ & \quad g_{9} \left( {\overline{x}} \right) = 0.515 - f_{0} \le 0{,} \\ {\text{where}}, & \quad f_{c} = 37.91\left\{ {1 + \left\{ {1.04\left( {\frac{1 - \gamma }{{1 + \gamma }}} \right)^{1.72} \left( {\frac{{f_{i} \left( {2f_{0} - 1} \right)}}{{f_{0} \left( {2f_{i} - 1} \right)}}} \right)^{0.41} } \right\}^{10/3} } \right\}^{ - 0.3} , \\ & \quad \gamma = \frac{{D_{b} {\text{cos}}\left( \alpha \right)}}{{D_{m} }},f_{i} = \frac{{r_{i} }}{{D_{b} }},f_{0} = \frac{{r_{0} }}{{D_{b} }}, \\ & \quad \phi_{0} = 2\pi - 2 \times {\text{cos}}^{ - 1} \left( {\frac{{\{ \left( {D - d} \right)/2 - 3\left( {T/4} \right)\}^{2} + \left\{ {D/2 - \left( {T/4} \right) - D_{b} } \right\}^{2} - \{ d/2 + \left( {T/4} \right)\}^{2} }}{{2\left\{ {\left( {D - d} \right)/2 - 3\left( {T/4} \right)} \right\}(D/2 - \left( {T/4} \right) - D_{b} \} }}} \right), \\ & \quad T = D - d - 2D_{b} ,D = 160,d = 90,B_{w} = 30, \\ {\text{Parameters range}}: & \quad 0.5\left( {D + d} \right) \le D_{m} \le 0.6\left( {D + d} \right),0.5\left( {D + d} \right) \le D_{m} \le 0.6\left( {D + d} \right),4 \le Z \le 50, \\ & \quad 0.515 \le f_{i} \le 0.6,0.515 \le f_{0} \le 0.6,0.4 \le K_{Dmin} \le 0.5,0.6 \le K_{Dmax} \le 0.7, \\ & \quad 0.3 \le \le 0.4,0.02 \le e \le 0.1,0.6 \le \zeta \le 0.85. \\ \end{aligned}$$

The optimization results of different competitive algorithms for the rolling element bearing design problems are presented in Table. 21 . What can be clearly seen is that the optimization results of TEAO and SO simultaneously produce the optimal cost of 1.70e + 04 and different solutions.

**Table 21:** Comparison results for the rolling element bearing design problem.

Algorithm	Optimal values for Parameters										Optimal value	Ranking
	***x***_***1***_	***x***_***2***_	***x***_***3***_	***x***_***4***_	***x***_***5***_	***x***_***6***_	***x***_***7***_	***x***_***8***_	***x***_***8***_	***x***_***10***_		
AO	1.26E + 02	1.80E + 01	4.65E + 00	6.00E-01	6.00E-01	5.00E-01	6.15E-01	3.04E-01	5.70E-02	6.00E-01	1.71E + 04	13
GWO	1.30E + 02	1.80E + 01	5.04E + 00	6.00E-01	6.00E-01	4.31E-01	6.80E-01	3.34E-01	4.10E-02	6.00E-01	1.70E + 04	6
CPSOGSA	1.29E + 02	1.80E + 01	4.70E + 00	6.00E-01	6.00E-01	4.02E-01	6.26E-01	3.15E-01	3.29E-02	6.00E-01	1.70E + 04	7
GJO	1.27E + 02	1.80E + 01	4.87E + 00	6.00E-01	6.00E-01	4.01E-01	7.00E-01	3.99E-01	2.43E-02	6.00E-01	1.70E + 04	10
WOA	1.25E + 02	1.80E + 01	4.51E + 00	6.00E-01	6.00E-01	5.00E-01	7.00E-01	3.00E-01	1.00E-01	6.00E-01	1.71E + 04	12
AVOA	1.29E + 02	1.80E + 01	4.97E + 00	6.00E-01	6.00E-01	4.95E-01	6.97E-01	3.58E-01	9.75E-02	6.00E-01	1.70E + 04	8
SSA	1.29E + 02	1.80E + 01	5.22E + 00	6.00E-01	6.00E-01	4.90E-01	6.74E-01	3.02E-01	3.82E-02	6.00E-01	1.70E + 04	9
RUN	1.30E + 02	1.80E + 01	4.51E + 00	6.00E-01	6.00E-01	4.04E-01	6.39E-01	3.22E-01	2.10E-02	6.00E-01	1.70E + 04	4
HHO	1.31E + 02	1.80E + 01	4.56E + 00	6.00E-01	6.00E-01	5.00E-01	6.00E-01	3.00E-01	9.58E-02	6.00E-01	1.70E + 04	3
SO	1.31E + 02	1.80E + 01	4.51E + 00	6.00E-01	6.00E-01	4.00E-01	7.00E-01	3.00E-01	1.00E-01	6.00E-01	1.70E + 04	1
PSO	1.63E + 03	9.06E + 02	1.41E + 03	5.15E-01	1.10E + 02	2.57E + 01	3.18E + 02	1.61E + 02	1.30E + 02	9.06E + 01	1.16E + 08	15
LPB	1.30E + 02	1.80E + 01	4.75E + 00	6.00E-01	6.00E-01	4.09E-01	7.00E-01	3.25E-01	8.37E-02	6.00E-01	1.70E + 04	5
FOX	1.25E + 02	1.80E + 01	4.51E + 00	6.00E-01	5.94E-01	4.00E-01	7.00E-01	3.00E-01	8.54E-02	6.00E-01	1.72E + 04	14
DBO	1.25E + 02	1.80E + 01	4.51E + 00	6.00E-01	6.00E-01	4.54E-01	7.00E-01	3.94E-01	2.00E-02	6.00E-01	1.71E + 04	11
**TEAO**	**1.31E + 02**	**1.80E + 01**	**4.51E + 00**	**6.00E-01**	**6.00E-01**	**5.00E-01**	**7.00E-01**	**3.00E-01**	**9.36E-02**	**6.00E-01**	**1.70E + 04**	**1**

### Gear train design (GTD)

**Figure 16:**
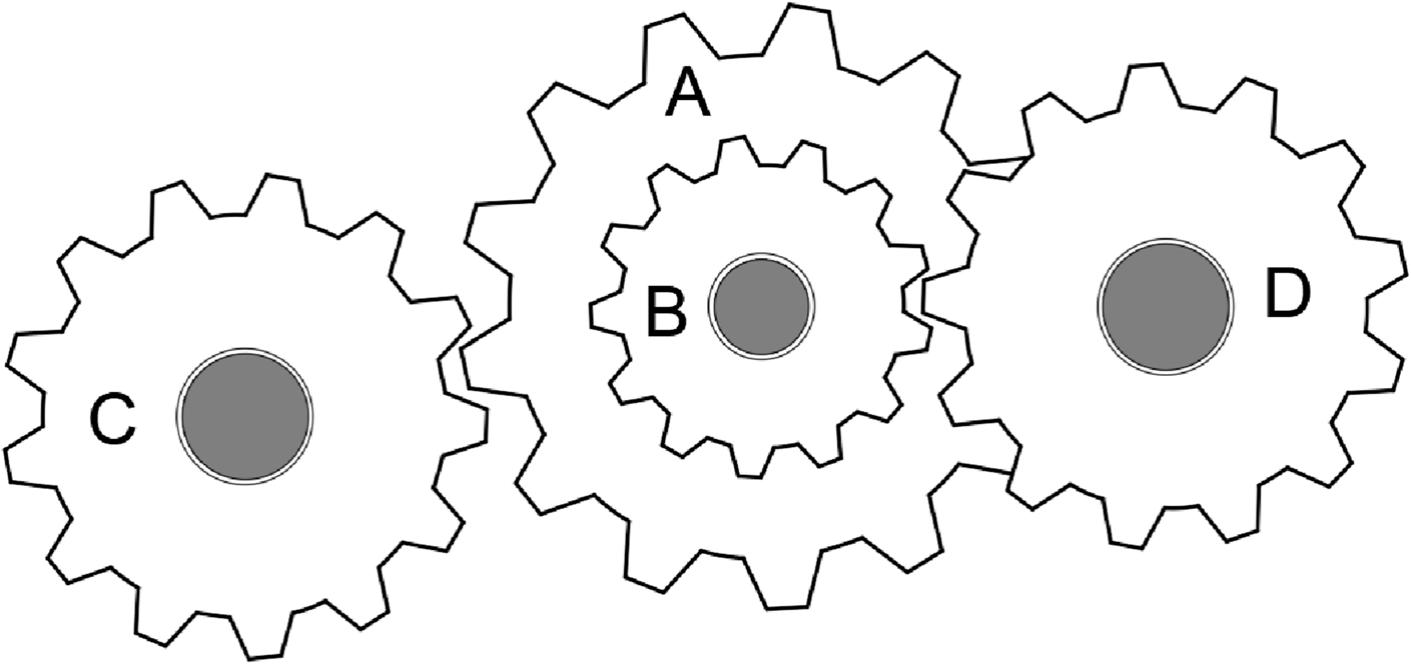
Gear train structure diagram.

The gear train design problem is a practical issue in the field of mechanical engineering. The objective is to minimize the ratio of output to input angular velocities of the gear train by designing relevant parameters of the gears. Figure 16 provides the structure of the optimization problem, and Eq. (32) describes the mathematical model for the optimization problem.

32 $$\begin{aligned} {\text{Consider}}, & \quad \vec{x} = \left[ {x_{1} x_{2} x_{3} x_{4} } \right] = \left[ {n_{A} n_{B} n_{C} n_{D} } \right] \\ {\text{Minimize}}, & \quad f\left( {\vec{x}} \right) = \left( {\frac{1}{6.931} - \frac{{x_{1} x_{2} }}{{x_{3} x_{4} }}} \right)^{2} , \\ {\text{Parameter range}}: & \quad 12 \le x_{1} ,x_{2} ,x_{3} ,x_{4} \le 60. \\ \end{aligned}$$

As can be seen from Table 22, TEAO, WOA, and HHO simultaneously obtain the minimum design cost of gear train design of 0.00E + 00 and produce different solutions.

**Table 22:** TEAO designs experimental results in gear train.

Algorithm	Optimal values for Variable				Optimal value	Ranking
	***x***_***1***_	***x***_***2***_	***x***_***3***_	***x***_***4***_		
AO	1.34E + 01	2.69E + 01	4.82E + 01	5.20E + 01	2.22E-10	15
GWO	1.46E + 01	1.42E + 01	3.93E + 01	3.65E + 01	8.76E-16	10
CPSOGSA	1.56E + 01	1.20E + 01	3.40E + 01	3.80E + 01	1.32E-28	6
GJO	3.66E + 01	1.20E + 01	5.10E + 01	5.97E + 01	1.20E-12	13
WOA	1.41E + 01	1.90E + 01	5.03E + 01	3.70E + 01	0.00E + 00	1
AVOA	1.33E + 01	1.46E + 01	2.87E + 01	4.67E + 01	2.61E-29	5
SSA	1.20E + 01	3.06E + 01	5.99E + 01	4.25E + 01	1.64E-21	8
RUN	1.39E + 01	3.19E + 01	6.00E + 01	5.12E + 01	1.04E-17	9
HHO	1.85E + 01	1.85E + 01	4.48E + 01	5.32E + 01	0.00E + 00	1
SO	1.89E + 01	1.20E + 01	5.12E + 01	3.07E + 01	4.10E-13	12
PSO	1.36E + 01	1.86E + 01	2.93E + 01	5.96E + 01	9.39E-28	7
LPB	1.20E + 01	2.16E + 01	4.19E + 01	4.29E + 01	1.65E-13	11
FOX	3.25E + 01	1.60E + 01	6.00E + 01	6.00E + 01	1.25E-10	14
DBO	1.20E + 01	1.20E + 01	3.27E + 01	3.06E + 01	0.00E + 00	1
**TEAO**	**1.38E + 01**	**2.63E + 01**	**6.00E + 01**	**4.19E + 01**	**0.00E + 00**	**1**

**Figure 17:**
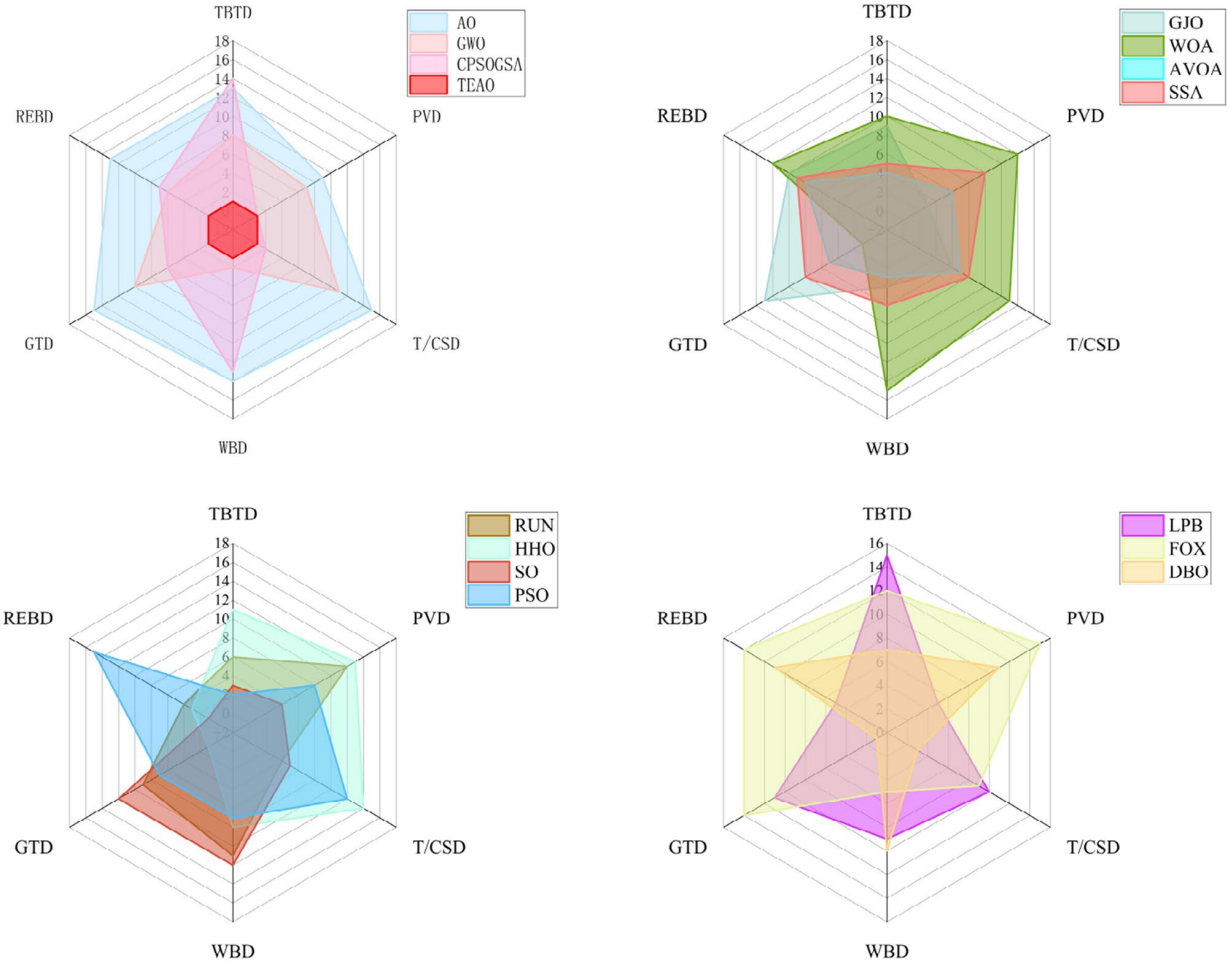
Comparison of experimental results of different algorithms in 6 engineering problems.

In sections "Three-bar truss design (TBTD)" to 5.6, we conducted comparative validations of TEAO and AO against thirteen other advanced algorithms on six real engineering problems. To showcase the comparative performance, we employed radar charts to visualize the rankings of each algorithm across different engineering problems, as depicted in Fig. 17. A smaller area in the chart represents better performance across the ten engineering problems. From the chart, it is evident that TEAO achieved the optimal solution in every engineering problem, clearly indicating not only outstanding performance but also high stability in solving real-world problems. The experiments in this section have thoroughly demonstrated the versatility and scalability of the TEAO method, laying a solid foundation for its practical application in engineering problems.

## Conclusion and prospects

In this paper, we propose an enhanced AO (TEAO) that utilizes a Tent chaotic map for initializing the population and incorporates new update rules. Addressing issues such as uneven distribution, significant randomness, limited capacity to handle optimization problems, and slow convergence of the original AO population, we introduce the Tent chaotic map for population initialization. Additionally, we present new exploration and exploitation formulas to enhance the algorithm's convergence speed without compromising accuracy.

To assess the performance of TEAO, we initially subjected it to testing using 23 benchmark functions. The experimental results demonstrate that TEAO exhibits higher accuracy and faster convergence speed compared to other algorithms used for comparison. Subsequently, we tested TEAO on 13 benchmark functions to evaluate its performance in handling problems with varying dimensions. The results indicate that TEAO effectively addresses optimization problems and displays robustness. Finally, we applied TEAO to solve six classical engineering design problems. The comparison with AO and 14 other algorithms further substantiates that the TEAO proposed in this paper holds a distinct competitive advantage.

Moving forward, our plan is to apply TEAO to address additional practical problems, including (1) workshop scheduling, (2) image processing, and (3) robot path planning.

## Data Availability

All data generated or analysed during this study are included in this published article.
